# Marine Lipids and Alzheimer’s Disease: Biochemistry, Bioaccessibility/Bioavailability, Metabolism, and Health Effects

**DOI:** 10.3390/md24060197

**Published:** 2026-06-03

**Authors:** Ana Gomes-Bispo, Carlos Cardoso, Cláudia Afonso, Helena Maria Lourenço, Sónia Pedro, Patrícia Moniz, Narcisa M. Bandarra

**Affiliations:** 1 Division of Aquaculture, Upgrading and Bioprospection (DivAV), Portuguese Institute for the Sea and Atmosphere (IPMA, I.P.), Av. Alfredo Magalhães Ramalho, 6, 1495-165 Algés, Portugal; 2 Interdisciplinary Centre of Marine and Environmental Research (CIIMAR), University of Porto, Rua dos Bragas 289, 4050-123 Porto, Portugal

**Keywords:** marine lipids, Alzheimer’s disease, phospholipids, sterols, terpenoids

## Abstract

Due to its high prevalence and significant impact on modern society, Alzheimer’s disease (AD) is one of the most important neurodegenerative disorders. It is more common among individuals over the age of 65, and its incidence has increased sharply as a result of rising life expectancy. Several factors have made it challenging to identify an effective treatment for AD. One major difficulty lies in its complexity, as the mechanisms involved in its progression are not yet fully understood. Nevertheless, the role of diet and lipids has been highlighted by numerous studies, underscoring their potential influence on this pathology. Due to the intricacy of its biochemical and metabolic interactions, this subject continues to be of particular interest, highlighting the need for further research. In this sense, this comprehensive and updated review aimed to elucidate these aspects, especially regarding marine-derived lipids, whose bioactive potential may become an irreplaceable tool in the management of AD, whether in terms of its treatment or prevention.

## 1. Introduction

Marine organisms are a very diverse group from both taxonomical and biochemical points of view [1]. There are micro- and macroalgae (seaweed), marine invertebrates and vertebrates, marine bacteria, and many other organism groupings. In addition, the marine environment is an invaluable source of diverse natural molecules with a huge variety of biological activities. The chemical diversity associated with these molecules is remarkable, comprising alkaloids, halogenated compounds, lactones, lipids, nucleosides, peptides, phenolics, polyketides, etc., some of which have very uncommon structural features. This reality is also matched by a great variety of metabolic pathways among sea species, including those leading to the synthesis of lipids and lipophilic molecules. In fact, marine lipids, in turn, comprise several vast compound classes, ranging from phospholipids [2] to glycolipids [3], and sterols [4] to terpenoids [5], among others.

This plethora of molecules includes some that have specific biological activities that may have positive health effects. Namely, there have been numerous reported instances of antibacterial [6], antioxidant [7], anticancer [8], anti-inflammatory [9], anti-hypertensive [10], and antidiabetic [11] activities. Accordingly, interest in marine organisms for the development of new drugs, nutraceuticals, and dietary supplements has been growing. Moreover, there are studies supporting a positive effect of marine-derived molecules on learning and memory function in neurodegenerative conditions [12]. In particular, the neuroprotective action of key bioactive compounds from some seaweed species, such as fucoidan, racemosins or dioxinodehydroeckol and other phlorotannins, has been reviewed by Alghazwi et al. [13]. The main in vitro bioactivities of these compounds comprise inhibition of acetylcholinesterase (AChE) and butyrylcholinesterase (BChE), inhibition of the amyloidogenic pathway (in which amyloid precursor protein (APP) is cleaved leading to beta amyloid [Aβ] peptides accumulation), anti-inflammatory activity, reduction in oxidative stress, kinase inhibition or the reduction in dopaminergic neurotoxicity [13].

Among neurodegenerative conditions (Alzheimer’s disease [AD], amyotrophic lateral sclerosis, Huntington’s disease, multiple sclerosis, Parkinson’s disease), AD has particular importance and widespread presence, being more prevalent in aged societies [14]. This neurodegenerative disease has a distinctively progressive nature, ultimately leading to a severe cognitive decline. Though early-onset forms are reported in the literature (around 10% of all AD cases), AD is more frequent among older people (>65 years old) [15]. The absence of treatment for AD is related to the insufficient understanding about the specific molecular mechanics underlying pathogenesis [14]. Its etiology remains a not-fully-understood subject. Though excessive accumulation of Aβ peptides is the main responsible for the progressive loss of neurons, the association of AD to the genes related to Aβ peptides formation or Tau hyperphosphorylation (excessive accumulation of phosphate groups in Tau protein) does not enable to encompass all the complexity of AD and neither provides a truly effective target for treatment [14]. In fact, those treatments targeting the Aβ peptides accumulation have been unable to treat or prevent AD. Indeed, based on the Clinicaltrials.gov platform, 413 AD trials were conducted between 2002 and 2012, and more than 99% of the drug candidates targeting amyloid pathways, comprising β or γ secretase inhibitors and Aβ peptides itself, are unable to significantly contribute to the fight against AD [14,16]. Moreover, some authors have suggested the existence of different biological subtypes of AD [17]. Hence, the current state of the art concerning AD points to a “multifactorial pathology” and highlights the importance of considering the complex interactions between different pathways. Dietary choices may have also a bearing on AD, being claimed, for instance, that a Mediterranean diet may be a beneficial approach in slowing the progression of AD [18]. Marine nutrients with biological activities, such as those mentioned above (AChE inhibition, Aβ peptides inhibition or anti-inflammatory activity), may give a meaningful contribution against AD.

In particular, numerous epidemiological studies have highlighted close relationships between AD incidence and pathogenesis and dietary lipids [19]. It is assumed that the maintenance of appropriate cell membrane lipid content could protect neurons, being n-3 polyunsaturated fatty acids (n-3 PUFA) of particular interest in the prevention of AD, since these n-3 PUFA are major constituents of neuronal lipids [19]. On the other hand, it has been argued that there is insufficient available evidence to advise the use of any primary prevention therapy for AD. This includes long-chain n-3 PUFA primary therapy, encompassing both eicosapentaenoic (EPA, 20:5 n-3) and docosahexaenoic acid (DHA, 22:6 n-3) [20], found in fish and some dietary supplements [21]. It has been suggested that long-chain n-3 PUFA content may not be the only protective component of seafood, as other nutrients and bioactive compounds, including marine antioxidants such as astaxanthin, selenium, vitamins (A, D, B12), minerals (iodine, zinc), and various marine polysaccharides and glycolipids, also likely contribute to the neuroprotective effects of seafood [22,23,24]. Some studies claim that there is no protective effect of these lipid molecules on prospective cognitive decline [25,26]. Such discrepancies between studies have been ascribed either to the choice of biomarker, apolipoprotein E type 4 allele (APOE ε4) genotype of the study participants or the particular kind of fish intake [20,27,28].

Therefore, the possible association between marine lipids and the onset and progression of AD is a major scientific subject that is not fully understood and, as such, remains partially controversial, thus warranting further study on all the biochemical and metabolic aspects and their repercussions on health and justifying a comprehensive and updated review of these debatable themes and also of related matters, such as the bioprospecting of key compounds and their bioaccessibility and bioavailability, or the food-nutraceutical dichotomy.

### Literature Search Procedure

Since the current review aimed to provide an all-encompassing perspective concerning marine lipids and AD, that is, ranging from the biochemical characterization of different groups of biomolecules in the biomass of marine organisms to the elucidation of the possible mechanisms that trigger the onset of the disease and its progression and may be thwarted, a broad, intensive, well-structured, and systematic literature search was required and, as such, it was carried out. In this search some crucial aspects were considered: (i) the major assumption that all studies regardless of being observational or randomized controlled trials should be encompassed; (ii) a balanced approach covering studies with different levels of evidence; (iii) a broad understanding of lipid biomolecules ranging from phospholipids to terpenoids and other groupings; and (iv) special attention to the representativeness of the main marine taxonomic groupings. Given the challenge’s dimension, a literature search methodology was designed. For this, a structured literature search was performed to ensure a comprehensive overview of the selected marine lipids and their potential relevance to AD.

This approach comprised four phases: (i) establishment of a list of subjects (marine lipids in general, phospholipids, glycolipids, sterols, terpenoids, neurodegenerative disease in general, AD pathogenesis and associated mechanisms, AD prevention and treatment, AD and dietary aspects) to be used as search themes; (ii) performance of the search itself in adequate media, defining in advance the questions to be addressed, the terms to be used in the search, the criteria of inclusion and exclusion, the decision tree to be applied during the process of including or excluding studies and the workflow to be followed during the literature search; (iii) gleaning of the relevant information of the multiple sources and its hierarchization; and (iv) comparative and critical analysis of the information for its utilization in this review. For the literature search, keywords (including specific compounds and groups of marine organisms, AD biomarkers, and terms associated with mechanisms known to have some connection to AD) were identified and combined using Boolean operators (AND/OR). Keywords and controlled terms like “marine lipids,” “phospholipids,” “glycolipids,” “sterols,” “terpenoids,” “Alzheimer’s disease,” “neurodegeneration,” “amyloid-β,” “tau,” and “oxidative stress,” are some examples. Both experimental and observational studies, including in vitro, in vivo, and clinical research, were considered in order to capture a broad spectrum of evidence.

Studies were selected based on their relevance to the topic, scientific quality, and contribution to understanding the relationship between marine lipids and AD, followed by critical analysis and synthesis of the extracted information.

Among the explored databases were: Sciencedirect, PubMed, Google Scholar, as well as relevant publishing houses, such as Taylor & Francis or Springer. There was also a general web search (including official entities, such as the World Health Organization). Search also included all sorts of scientific/technical publications (papers, books, theses, etc.) both in Portuguese and English.

## 2. Marine Resources and Their Biomolecule Trove

Marine lipids comprise a large group of different compounds, ranging from triacylglycerols (TAGs) to glycerophospholipids (PLs), glycolipids, free fatty acids (FFAs), and other more specific molecules. In a broad sense, terpenoids and other eminently lipophilic substances may be included under the definition of marine lipids. On the other hand, some molecules are simple nutrients, constituting basically a source of energy—such as the TAG—and not displaying significant bioactivity. Accordingly, the authors of this review chose to focus on the most bioactive groupings and to structure the study along three main axes: (i) phospholipids; (ii) terpenoids and (iii) sterols. These three large classes are all well represented in the biomass of marine organisms [2,3,4,5].

### 2.1. Phospholipids

**Figure 1:**
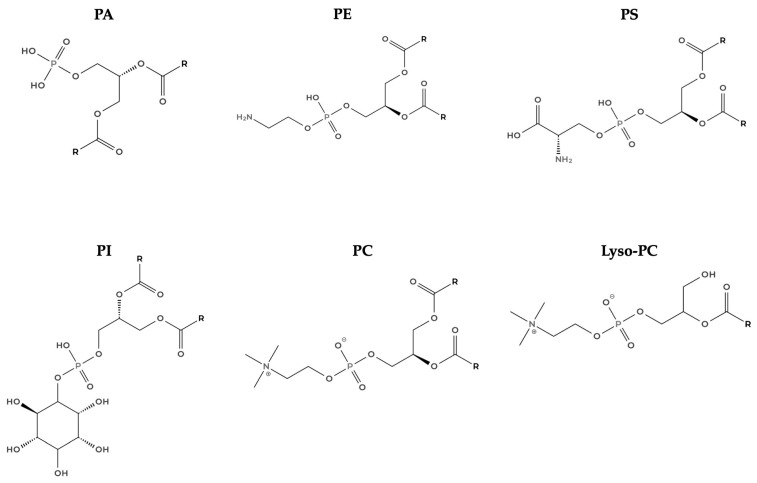
Generic chemical structures of the most important glycerophospholipids: phosphatidic acid (PA), phosphatidylethanolamine (PE), phosphatidylserine (PS), phosphatidylinositol (PI), phosphatidylcholine (PC) and lyso-phosphatidylcholine (Lyso-PC).

**Figure 2:**
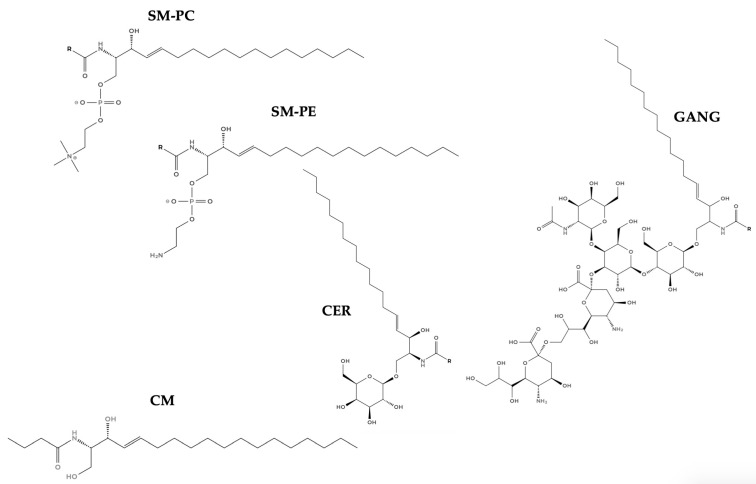
Generic chemical structures of the most important sphingophospholipids (SPLs): sphingomyelin (SM), phosphatidylcholine (PC), phosphatidylethanolamine (PE), cerebroside (CER), ceramide (CM), disialoganglioside GD2 (GANG).

The PL can be subdivided into two main groupings: PL and sphingophospholipids (SPLs) [29]. The former grouping is constituted by fatty acids (FAs) esterified to a glycerol backbone and a phosphate group linked to this backbone and to a hydrophilic residue, such as choline, resulting in phosphatidylcholine (PC), serine, producing phosphatidylserine (PS), inositol, yielding phosphatidylinositol (PI), and ethanolamine, resulting in phosphatidylethanolamine (PE) (Figure 1). If none of these residues are attached, then the simpler molecule is phosphatidic acid (PA). Lysophospholipids (Lyso-PLs) are PL whose FA chain has been hydrolyzed from either the sn-1 or sn-2 position [30]. The alkyl-PL constitute another form of PL. The alkyl-PL are characterized by containing one FA in the sn-2 position and one aliphatic chain in the sn-1 position attached to the glycerol backbone by an ether bond, plasmalogens (PLGs) being the most common example [2]. Plasmalogens possess a specific vinyl ether bond. The latter grouping, SPL, has its backbone formed by the long-chain amino-alcohol sphingosine instead of glycerol, sphingomyelin (SM) being a representative example that is present at high levels in the brain and neural tissue and consists of sphingosine esterified to a phosphocholine group as well as a FA (Figure 2). Cerebroside (CER) results from the condensation reaction of a ceramide (CM) and a single sugar molecule. Just as CER, gangliosides result from the condensation reaction of CM and carbohydrate molecules. However, gangliosides comprise multiple combinations of oligosaccharides and one or more sialic acid component(s), such as exemplified in Figure 2 by the disialoganglioside GD2 (GANG). Both PL and SPL are constituents of biological membranes.

It should be remarked that the overwhelming majority of the FA are found in the form of TAG [31], followed by PL, diacylglycerols (DAGs), cholesterol esters, and lipid-soluble vitamin esters, such as retinyl palmitate and tocopherol acetate. All these lipid classes display significantly different levels of bioaccessibility/bioavailability [32]. In addition, FA delivered in the form of PL are considered to be more efficiently bioactive than TAG due to their higher uptake by the brain [33]. Namely, krill (an important marine resource) oil contains approximately 35% of its DHA in the form of PL, being reported to be more bioactive and bioavailable than oils whose FA are found in the form of TAG [34].

This class of lipids, PL, may be quite rich in PUFA. In accordance with their amphiphilic biochemistry, naturally occurring PL generally have an unsaturated FA (very frequently PUFA) in the sn-2 position, such as EPA or DHA, whereas the sn-1 position frequently carries a saturated fatty acid (SFA), such as palmitic (16:0) or stearic (18:0) acids [30]. In marine sources, the n-3 PUFA PL, defined as PL containing n-3 long-chain PUFA, are quantitatively very important, making marine sources distinctive from other biological sources [35]. In fact, seafoods are considered to be the main food source of n-3 PUFA with all potential health benefits associated with EPA and DHA [33], including prevention of AD [19]. In general, marine PL may be very rich in both n-3 long chain PUFA.

These PL may have multiple functions in the living organism. Their bound PUFA can be released on demand as precursors of prostaglandins and other eicosanoids [36], while other PL molecules and corresponding metabolites are a starting material for the biosynthesis of secondary messengers in cell signalling (e.g., phosphoinositides) [37], performing essential tasks within mitochondria [38] and other organelles. Moreover, PA also acts as a signalling lipid, assisting in the recruitment of cytosolic proteins (e.g., sphingosine kinase) to the correct membranes [39]. Hence, besides being integral structural lipids in the cell membranes, PL have also been reported to perform many other roles in various cell types and organisms [30].

The marine resources contain a vast variety of PL compounds, thus constituting a biomolecule trove and an excellent source of bioactive PUFA. A recent study on the pelagic fish Pacific saury (*Cololabis saira*) [40] has reported that the major components of its PL fraction were PC (89 mg/g of the PL fraction), PI (53 mg/g), and PE (40 mg/g), while PS, Lyso-PC, and Lyso-PE were the minor components at 5, 15, and 19 mg/g, respectively. DHA and palmitic acid were the major FAs [40]. The analysis of the major PL groups in krill (*Euphausia superba*) yielded 80% PC, 15% PE, and only 1% PI [41]. Hwang, et al. [42] analyzed the lipid composition of the integument belonging to the squid *Dosidicus gigas* and concluded that it included more than 70% of PL, which were composed mainly by PC (about 45% of total PL), PE (23%), and PS (7%). It should be remarked that the integument is a squid processing co-product that is separated from the edible part when unfrozen by hot water, being treated as a fishery waste in a perspective of circular economy and zero waste [43]. Other authors [44] were able to extract EPA-rich PC from the sea cucumber *Cucumaria frondosa* and DHA-rich PC from squid roe for studying their anti-AD potential. Squid roe has been used as privileged source of marine PL compounds, for instance, a mixture of DHA-PC and DHA-PS was attained from the roe of the purpleback flying squid *Sthenoteuthis oualaniensis* by Wang et al. [45]. Wen, et al. [46] also used this species’ roe for preparing DHA-PC and DHA-PS. Besides roe, ovaries of marine organisms may also be excellent sources of bioactive PL molecules, for instance, DHA-PL from the ovary of skipjack (*Euthynnus pelamis*), a kind of tuna, was extracted and tested [47]. There are also meaningful examples involving PLG, such as DHA-rich ethanolamine plasmalogen (EtnPLG) that was purified from the viscera of an ascidian (a marine invertebrate that is a filter feeder) by Yamashita et al. [48] and EPA-EtnPLG from sea cucumber (*Calceolaria frondosa*) [49]. Within the domain of the less-known marine organisms, Standal, et al. [50] reported the detailed composition of the PL in the ragworm *Alitta virens*, an annelid, by ^31^P nuclear magnetic resonance. The composition described had several different PL species: PC accounted for 44.9 ± 0.8%, followed by PE (20.5 ± 0.9%), and Etn/SM PLG (8.5 ± 0.7%). Less-pure forms of PLG have also been extracted and used in experiments [51]. Indeed, this study tested PLG with a mixture of FA (26.1% EPA and 28.7% DHA) derived from scallop, a bivalve mollusc.

Seaweed species also contain anti-inflammatory (nitric oxide [NO] inhibition against lipopolysaccharide (LPS)-induced NO production in RAW 264.7 cells) active PL, such as 1-*O*-eicosapentaenoyl-2-*O*-trans-3-hexadecenoyl-3-phospho-(10-glycerol)-glycerol (*trans* FA are uncommon) and 1-*O*-eicosapentaenoyl-2-*O*-palmitoyl-3-phospho-(10-glycerol)-glycerol in the ethyl acetate fraction of a methanol/chloroform extract from the red seaweed *Palmaria palmata* [52].

Hence, marine PL sources are widely variable from a taxonomic point of view. There are also important differences between relatively similar species; for instance, the shrimps *Penaeus chinesis* and *Macrobranchium nipponense* were reported to contain higher unsaturated fatty acyl compositions (monounsaturated fatty acids [MUFA], and PUFA) in PC molecules than *E. superba* and *M. rosenbergii* [53].

There are abiotic parameters that affect the concentration of particular PL in seafood tissues. In fact, it has been stated that from cold zones at high latitude to subtropical zones at low latitude the concentration of DHA-PC and DHA-PE in fish brains displays a decreasing trend [54]. Farmed fish seem to not exhibit such a trend, since they are reared on feed-controlled conditions and not fully subject to environmental abiotic parameters. Furthermore, the content and types of DHA-PL also depend on the particular tissue of the marine organism. DHA in fish brain is mainly present in the form of DHA-PC and DHA-PE [54]. A study on Pacific blue mackerel (*Scomber australasicus*) concluded that roe is the best source of marine n-3 PUFA PL among this fish species’ by-products [55]. In this case, the proportion of EPA in the sn-2 position was significantly higher in mackerel roe (12.6%) compared to the other by-products (head, 1.91%; skin, 2.22%; male gonad, 2.02%). In contrast, the level of DHA in the sn-2 position did not vary substantially among the various parts [55]. Additionally, the level of n-3 PUFA esterified in PL was higher in roe (55.5%) compared to head (40.9%), skin (21.8%), and male gonad (32.0%) [55].

The inclusion of seafood in many countries’ diets ensures a large role for this group of marine organisms in providing PL and, in particular, n-3 PUFA PL to the populations, but it should also be remarked that culinary treatment exerts an effect on the PL molecule composition of fish as food [56]. Deep-fat frying, particularly when using oils such as extra virgin olive oil or sunflower oils, leads to a marked reduction in PL species of long chain n-3 PUFA present in the seafood, especially those containing two DHA chains, while relatively increasing species composed of SFA or MUFA paired with PUFA [56]. This is largely due to the high susceptibility of DHA to heat- and oxidation-induced degradation, making DHA/DHA phospholipids more prone to breakdown during repeated frying compared to more stable combinations such as SFA/DHA pairs.

Given the role of PL compounds in metabolism and health, it may be important to extract natural PL molecules from the marine matrices. The whole PL fraction or particular PL substances may be the target. Such extraction is potentially advantageous because it enables us to attain very concentrated fractions that may be incorporated in food, medicines, and other applications afterwards. Furthermore, the extraction procedures may enable us to prepare fractions with tailored PL compositions, for instance, with a specific percentage of PC. Nonetheless, PL extraction carries equal disadvantages and problems. Namely, due to containing n-3 PUFA, marine PL compounds are highly prone to oxidation in conventional extractions performed by the incorporation of organic solvents, exposure to atmospheric oxygen, and high temperature [57]. Moreover, the organic solvents used to treat the crude PL fraction are considered detrimental due to negative impacts on health and environment [57]. An alternative approach is supercritical carbon dioxide (SC-CO_2_) extraction, as it operates under relatively mild conditions and avoids the use of large amounts of conventional organic solvents. SC-CO_2_ extraction has been reported to produce high-quality marine PL extracts [57]. However, despite these advantages, the method may also present limitations, including high equipment and operational costs, as well as variable extraction efficiency depending on the raw material and extraction conditions. Ali-Nehari and Chun [41] applied SC-CO_2_ extraction for krill PL recovery in a two-step process (SC-CO_2_ extraction followed by ethanol extraction) and optimized the extraction conditions at 45 °C, 25 MPa, and a CO_2_ flow rate of 22 g/min. Enzymatic-assisted processes may also represent promising alternatives in the future, although further research is still warranted.

It is also possible to synthesize novel PL either by using extant marine PL as raw material and introducing new biochemically relevant groups and other chemical changes or by taking clues and stereochemical ideas from natural molecules and synthesizing de novo pure PL without similar counterparts in nature. The synthesis of structured PL, in particular DHA-containing PL, is an example of this latter situation. For instance, Lagarde, et al. [58] were able to produce such a structured PL, 1-acetyl,2-docosahexaenoyl-glycerophosphocholine (AceDoPC)—a synthetic PL that has received growing attention [59]. Contrary to natural marine PL, this molecule presented the advantage of preventing the transfer of DHA from the sn-2 to the sn-1 position [58]. This is particularly important, since there is a ten-fold preference for 1-lyso,2-docosahexaenoyl-glycerophosphocholine to be more effectively taken up by the mammal brain as tested in rats [60]. Other authors [61] have also worked with structured PL.

In a more recent study, Gomes, et al. [62] developed fish-based extracts enriched with DHA-Lyso-PC, designed for use as dietary supplements. To extract PL from the muscle of Atlantic mackerel (*S. scombrus*), they used food-grade green solvent such as ethyl acetate and ethanol. The extracts were then hydrolyzed using *Rhizomucor miehei* lipase, which converted approximately 50% of the PC in the ethanolic extracts into Lyso-PC. When comparing the FA profile of PC in the original ethanolic extracts to that of Lyso-PC in the hydrolyzed extracts, the researchers observed a significant increase in the relative content of DHA—from 33.6% to 73.6% of total FA, representing a 40% increase [62]. Based on these findings, the authors concluded that the extracts—obtained from an underutilized fish species like *S. scombrus*—could serve as an effective strategy for the blue economy to improve DHA uptake in brain cells, while also contributing to the valorisation of undervalued species. This approach can be considered reasonable because Lyso-PL, such as Lyso-PC, are resistant to degradation by lipases and phospholipases during lipid digestion and metabolism. As a result, DHA-Lyso-PC can be transported across the blood–brain barrier and delivered directly to brain tissue [63].

The utilization of synthetic DHA-PS alongside DHA-PC has also been endeavoured [64]. These PL have been prepared from different sources, namely, L-serine and DHA-PC extracted from squid roe [64]. The enzymatic synthesis of DHA-PS is a transphosphatidylation of DHA-PC catalyzed by phospholipase D [65]. This enzymatic reaction leaves DHA at the same position; thus, if DHA is located at an sn-2 position, it will generate sn-2 DHA-PS. There may be some hydrolysis with formation of PA, for instance, [46] found 3.6% PA in the DHA-PS product, but all the remaining 96.4% is PS. Hence, this is a highly effective way to synthesize specific PS molecules. Similar synthesis procedures have been applied for attaining specific PE molecules [66]. Che, et al. [66] extracted EPA-PC from *C. frondosa* and, afterwards, synthesized EPA-PE by phospholipase D catalyzed transphosphatidylation of EPA-PC in an ethanolamine-containing buffer system.

Therefore, the discovery of novel marine PL molecules may pave the way for future nutraceuticals or further strengthen the case for a higher seafood consumption—namely particular seafood-rich in specific PL. In the former case, mild extraction processes must be prioritized and chemical or enzymatic PL synthesis for more effective and bioavailable using such molecules as templates is also a pertinent strategy.

### 2.2. Terpenoids

**Figure 3:**
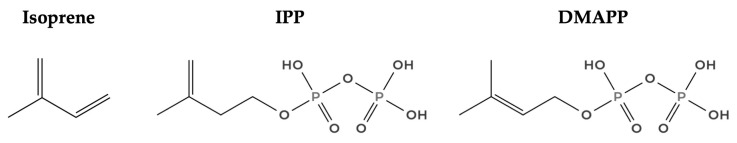
Isoprene, isopentenyl diphosphate (IPP) and dimethylallyl diphosphate (DMAPP) molecular structures.

Terpenoids comprise the most diverse group of natural compounds that represent nearly 60% of marine natural products [67], which are characterized by having isoprene as their basic unit (Figure 3). Terpenes are simple hydrocarbons, while terpenoids (or isoprenoids, how they are also called) are a modified class of terpenes with different functional groups. Nevertheless, the designation “terpenoids” is commonly used to describe both, terpenoids and terpenes. Terpenoids are formed by multiple isoprene units: isopentenyl diphosphate (IPP) and dimethylallyl diphosphate (DMAPP) (Figure 3), which are then assembled to each other through a wide variety of pathways, including the linear arrangement of isoprene units, cyclization and rearrangements of the carbon skeleton [68].

Due to their great variety, terpenoids are commonly classified according to their number of isoprene units and the number of carbon atoms as monoterpenoids, sesquiterpenoids, diterpenoids, triterpenoids, and tetraterpenoids (Table 1).

**Figure 4:**
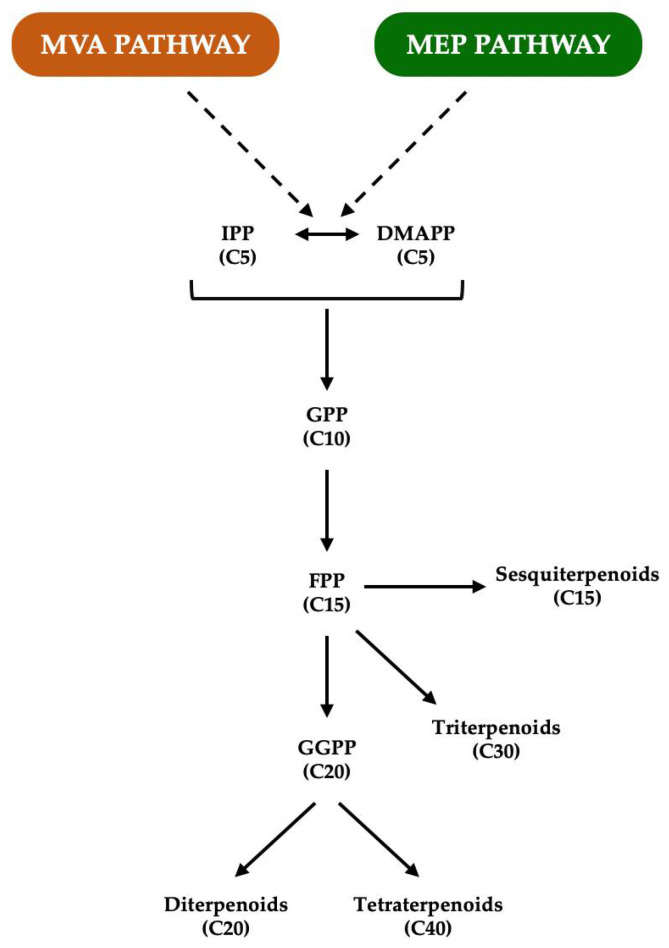
Biosynthesis of terpenoids through mevalonate (MVA) and 2-C-methyl-D-erythritol 4-phosphate (MEP) pathways and their respective intermediates: IPP, isopentenyl diphosphate; DMAPP, dimethylallyl diphosphate; GPP, geranyl pyrophosphate; FPP, farnesyl diphosphate; GGPP, geranylgeranyl diphosphate.

Despite their structural differences and irrespective of the pathway whence they originate from, these terpenoids have a common intermediate: IPP and DMAPP, which are interconvertible with each other. This isomerization is carried out by the action of the IPP isomerase. Actually, condensation of IPP and DMAPP is the starting point of terpenoids biosynthesis. In organisms, terpenoids are biosynthesized both through two main routes: the mevalonate (MVA) pathway and the 2-C-methyl-D-erythritol 4-phosphate (MEP) pathway (also designated as non-mevalonate pathway) (Figure 4). The former comprises a series of chain reactions that converts acetyl CoA into mevalonate-5-diphosphate which, ultimately, is transformed in IPP. This pathway that takes place in cytosol is the same through which cholesterol is produced [67]. In turn, the MEP is active in the plastids having pyruvate and glyceraldehyde-3-phosphate as the IPP precursors [69]. For this reason, in animals, the biosynthesis of isoprenoids is only possible through the MVA pathway, while algae are usually able to carry out both pathways. Still, some exceptions can occur, as it happens with green algae that only possess the MEP [69]. Among marine animals, molluscs, for example, are known to have high amounts of terpenoids. The presence of these compounds can result directly from the diet or its biotransformation, or from their de novo biosynthesis ability [67]. These compounds are used, for example, as a mechanism of defence of the marine organisms against predators, due to their repulsive or toxic properties [70,71,72].

Terpenoids biosynthesis is mediated by the action of the enzyme’s terpene synthases, that is followed by further secondary transformations, including the oxidation, reduction, isomerization, and conjugation reactions of the basic parent skeletons, which provide specific functional properties to the terpenoids [73,74]. Even though some exceptions may occur, the mono-, di-, and tetraterpenoids are mainly biosynthesized through the MEP pathway, while the sesqui- and triterpenoids are formed by the MVA pathway [68,75]. Then, sequential IPP fusions originate the geranyl pyrophosphate (GPP, C10), the farnesyl diphosphate (FPP, C15), and the geranylgeranyl diphosphate (GGPP, C20), which are the respective precursors of mono-(C10), sesqui-(C15), and diterpenoids (C20) (Figure 4). In turn, triterpenoids (C30) and tetraterpenoids (C40) are formed by head-to-head condensation of sesquiterpenoids and diterpenoids, respectively (Figure 4). While the enzymes belonging to the prenyltransferase family are responsible for the sequential incorporation of IPP units, the terpenoid synthases take part in terpenoids biosynthesis transforming the common prenyl diphosphate substrates, like GPP and GGPP, into the core scaffolds of numerous structurally distinct terpenoid groups [73]. Even though they catalyze a wide array of reactions, this family of enzymes exhibit a great specificity in terms of the lengths of isoprenoid backbone that is used as substrate [76].

**Figure 5:**
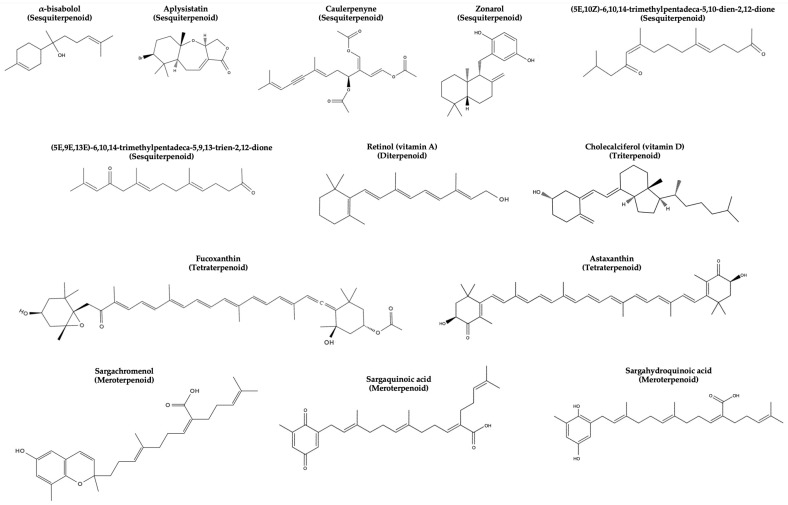
Molecular structures of relevant terpenoids found in marine resources.

Throughout recent years, an intense scientific investigation has allowed the discovery of numerous marine algae species that could be used as potential sources of new anti-AD substances. Most of these bioactives have been identified in seaweeds. For example, marine red seaweeds (Rhodophyta) were found to produce highly brominated and chlorinated terpenoids [70] with recognized anti-AD activity. This is the case of the halogenated monoterpenes (both bromide and chlorinated species) found in *Ochtodes secundiramea* [77] or the aplysistatin (Figure 5), a brominated sesquiterpenoid, discovered in *Laurencia snackeyi* (Weber-van Bosse) Masuda, in *L. luzonensis* and in *L. obtusa* [78,79,80]. In the later species, De Nys, et al. [79] accounted for 1.69 μg/mg in the dry weight (DW) of aplysistatin in the seaweed thalli, while 1 g of *L. snackeyi* crude extract contained 96.5 mg [80]. Caulerpenyne (Figure 5), also a sesquiterpenoid that is considered as the major or one of the main secondary metabolites, has been identified in several marine green seaweed from the genus *Caulerpa* [81,82]. According to Jung, et al. [82] this brominated sesquiterpenoid is produced as a mechanism of defence exerting a deterrent effect, for instance, against sea urchins. The levels of caulerpenyne can vary depending on the species, the location, and the season. Relative contents between 0.2 and 1.9% DW were reported for *C. racemosa* species and *C. taxifolia*, respectively [82]. On the other hand, Box, et al. [83] in their study with three different species collected along Western Mediterranean coastlines, found that caulerpenyne corresponded to 7.29%, 5.47%, and 0.43% DW in the fronds of *C. prolifera*, *C. taxifolia* and *C. racemosa*, respectively. In this region, *C. racemosa* and *C. taxifolia* are both considered to be invasive species, meaning that the exploitation of these seaweeds could provide a sustainable source to obtain caulerpenyne.

Species from the Phaeophyceae class (brown seaweeds) represent one of the richest sources of terpenoids with potential anti-AD activity, including sesquiterpenoids, tetra-terpenoids, and meroterpenoids. These compounds are considered promising due to their ability to target multiple pathological mechanisms associated with AD, such as the inhibition of AChE and BChE, reduction in Aβ peptides accumulation, attenuation of neuroinflammation, and mitigation of oxidative stress, all of which are key contributors to neurodegeneration and cognitive decline.

Among the sesquiterpenoids, α-bisabolol, zonarol, and farnesyl acetone derivatives are among the most relevant compounds reported for their potential neuroactive effects. Alpha-bisabolol (Figure 5), also known as levomenol, is a monocyclic sesquiterpene alcohol identified in extracts of the marine brown seaweed *Padina gymnospora* collected from the Gulf of Mannar region in the Indian Ocean [84]. Sethupathy, et al. [85], through bioassay-guided fractionation of a methanolic extract of *P. gymnospora*, reported fractions containing relative abundances of α-bisabolol as high as 69%, highlighting this compound as a major constituent potentially associated with the observed biological activity.

Zonarol is a sesquiterpene containing a hydroquinone group in its structure (Figure 5). This characteristic gives zonarol antioxidant properties, taking advantage over other antioxidant molecules in mediating cytoprotection against oxidative damage [86]. Zonarol can be found in marine brown algae like the *Dictyopteris undulata*, found in Japan.

Despite this, among seaweeds, those from the genus *Sargassum* seem to be the most promising source of anti-AD compounds. So far, a range of sesqui-, mero-, and tetraterpenoids with pharmacological potential have already been isolated from *Sargassum* sp. Ryu and co-workers [87], in their investigation of the Korean *S. sagamianum*, isolated two sesquiterpenoids (farnesyl acetones) with anti-AD potential due to their anticholinesterase activity: (5E,10Z)-6,10,14-trimethylpentadeca-5,10-dien-2,12-dione (dihydromonofarnesylacetone) and (5E,9E,13E)-6,10,14-trimethylpentadeca-5,9,13-trien-2,12-dione (monooxofarnesylacetone) (Figure 5).

Retinol (Figure 5), commonly known as vitamin A, is a diterpenoid obtained via the conversion of carotenoids and retinoids [88]. Once ingested, retinol can be converted to its active forms, such as retinal, retinoic acid, and retinyl esters. Animals are not able to synthesize retinol de novo; for this reason, it should be obtained from the diet in the form of retinol, that is later converted to its bioactive derivative all-*trans* retinoic acid [89], or as a carotenoid which can be converted into retinal or apocarotenoids (which subsequently can be converted to retinoids) [90,91].

Microalgae like *Chlorella vulgaris* and *Tetraselmis* sp. can contain 132.15 and 2.2 μg/g DW of retinol, respectively [92,93]. In turn, for brown algae *Undaria pinnatifida* and *Laminaria japonica* (commercial names Wakame and Kombu), 2.17 and 4.81 μg/g DW of retinol respectively were the contents determined by Kolb, et al. [94].

Just like the other terpenoids already addressed, retinol can be found in marine algae; however, marine fish are the most relevant dietary sources of this diterpenoid. Indeed, fish, and especially fish liver oils, are known as excellent sources of vitamin A. It is noteworthy that these organisms may contribute significantly to the ingestion of retinol (diterpenoid), cholecalciferol (triterpenoid) or astaxanthin (tetraterpenoid). Indeed, fish and shellfish are traditionally considered to be excellent food sources of retinol. The oysters, and oily fish, such as sardines, herring, horse mackerel and tuna, are some examples of species with high contents of vitamin A [95]. Even in leaner species, like cod, high amounts of this vitamins can be found in the liver [96,97]. In tuna, for example, vitamin A contents, can be as high as 450 μg/100 g of the edible portion, in sprat (*Sprattus sprattus*) between 100 and 150 μg/100 g, and in mullet (*Mugilidae* spp.) between 45 and 47 μg/100 g [96]. Erkan, et al. [98] reported contents of 141 μg/100 g in horse mackerel (*Trachurus trachurus*) edible muscle. More recently, Reksten and colleagues [99] described the nutritional composition of several fish species sampled along Sri Lanka coastline, with interesting amounts of vitamin A, particularly in the smaller species with mean contents of 295 μg/100 g (determined in the whole fish, according the local dietary habits). In species studied from the Bangladesh Coast, the unicorn cod (*Bregmaceros mcclellandi*) stood out with the highest vitamin A content, 280 μg/100 g, in fillets with skin and bones as they are consumed by local populations (excluding the head, tail, and viscera) [100].

Triterpenoids comprise the group of compounds with 30 carbon atoms and six isoprene units, which are derived from the squalene biosynthetic pathway [68,101]. Triterpenes are formed by two C15 units linked head-to-head [102]. These compounds can present relatively complex cyclic structures, and can occur as alcohols, aldehydes or carboxylic acids, which differentiate them biologically [101]. In addition, triterpenoids have many active sites that can undergo glycosylation and be converted to saponins (triterpene glycoside).

Cholecalciferol (Figure 5), usually designated as vitamin D, is a fat-soluble vitamin from the group of secosterols, that participate in many physiological functions in the central nervous system, endowed with anti-AD activity [103,104]. Once in circulation, vitamin D undergoes hydroxylation in two moments: first in the liver and then in the kidneys, where it is converted to its active form, calcitriol (1α,25-(OH)_2_D_3_) [105]. Cholecalciferol can be biosynthesized in the skin by ultraviolet radiation, yet it must also be obtained through the diet, namely from fish. In this context, salmon (*Salmo salar*), is typically considered as a good dietary source of cholecalciferol. Vitamin D levels reported by Byrdwell, et al. [106] ranged between 7.80 and 32.45 µg/100 g in salmon available in the U.S. retail market. This wide variation was also confirmed by Jakobsen and her co-workers [107], who proved that significant differences in vitamin D contents can take place between wild and farmed salmon, and depending on the place where it is caught. The fillet from wild salmon caught in the Baltic Sea and the North Sea had 18.5 and 9.4 µg/100 g, respectively. In turn, these authors reported contents between 2.9 and 9.5 µg/100 g for farmed salmon.

High levels of cholecalciferol are also found in other salmonid species like the canned pink salmon (*Oncorhynchus gorbuscha*) (12.7–43.5 µg/100 g) [108]. Pelagic fish are also good sources of this vitamin. This the case of Atlantic herring (*Clupea harengus*) (11–27 µg/100 g), sprat (*S. sprattus*) (8.7–20 µg/100 g), Atlantic mackerel (*S. scombrus*) (2.1–18.9 µg/100 g), and Atlantic horse mackerel (*T. trachurus*) (28 µg/100 g) [96,109,110].

Carotenoids, with a linear C40 backbone, are the most common tetraterpenoids. Their chemical structure is formed by a polyene hydrocarbon chain with conjugated double bonds, which can donate electrons and is in the base of their high reducing potential. In global terms, they can be divided in two main classes: carotenes and xanthophylls [111]. The formers are characterized for being nonpolar hydrocarbons, whereas xanthophylls, like fucoxanthin, are polar oxygenated derivatives of carotenes [112]. Carotenoids are produced by plants and algae, but also by several bacteria and fungi, from two GGPP that are joined head-to-head [102].

Humans cannot synthesize carotenoids de novo; hence the required physiological levels exclusively depend on their dietary intake. In the literature it is possible to find works concerning anti-AD tetraterpenoids isolated from *Sargassum* sp.

Among carotenoids, fucoxanthin has a unique structure (Figure 5). Besides the typical polyene chain, this xanthophyll contains an allenic bond, an epoxide and a conjugated carbonyl group [113]. Indeed, some studies dealing with fucoxanthin anti-AD activity were already undertaken with extracts obtained from brown seaweeds *S. horneri* and *S. siliquastrum* [114,115,116]. Besides *Sargassum* sp., other marine organisms can be considered as potential sources of this carotenoid. The microalgae *Isochrisis galbana* and *Phaeodactylum tricornutum* are the best candidates; however, the brown seaweeds *L. japonica*, *U. pinnatifida*, and *Himanthalia elongata*, [111,117,118,119,120,121] are also known to contain fucoxanthin. In fact, it is known that fucoxanthin is the main carotenoid present in *I. galbana* and *P. tricornutum*. Matos, et al. [122] and Kim, et al. [123] have reported, respectively, contents of 6.10 and 6.04 mg/g DW in *I. galbana*. However, a higher amount (18.23 mg/g DW) was found in the marine *Isochrysis* aff. *galbana* by Kim, et al. [123]. Furthermore, the found levels of this xanthophyll in *P. tricornutum* corresponded to 15.71 mg/g DW [124]. However, fucoxanthin levels in this microalga can reach higher values, up to 24.2 mg/g DW [125]. Lower amounts of fucoxanthin are found in *U. pinnatifida* (0.87 mg/g DW) and *L. japonica* (0.12 mg/g fresh weight).

Astaxanthin is another xanthophyll with therapeutic potential to treat neurological disorders including those related with AD. Besides its polyene chain and the two terminal rings (common to all carotenoids and xanthophylls, respectively), astaxanthin’s chemical structure is characterized to also have hydroxyl and keto groups (Figure 5).

*Haematococcus pluvialis*, a freshwater microalga, is among the chief commercial sources of astaxanthin. Still, this red xanthophyll is also widely distributed among marine organisms, including algae (where it is synthesized), and marine animals like salmonids, trout and shrimp (as a result of these animals’ diet) [126]. Astaxanthin is the main carotenoid in fish like Atlantic salmon, rainbow trout, Arctic char and red bream, where it is responsible for the characteristic pink and red colour of their flesh [127].

Turujman, et al. [128] quantified astaxanthin in several wild salmonid species, reporting levels of 30.0–58.9 mg/kg in sockeye salmon (*Oncorhynchus nerka*) and 4.9–7.2 mg/kg in Atlantic salmon (*S. salar*). Actually, the levels of astaxanthin in salmonids can vary widely, where it can be as high as 38 mg/kg [129].

The supplementation with astaxanthin has found a prolific application in salmonid aquaculture, impacting, for instance in the pigmentation of salmon, trout, and sea bream [130]. Sigurgisladottir, et al. [131] observed an increase in astaxanthin levels of approximately 0.5 to 2.5 mg/kg in the Atlantic salmon muscle after supplementation with this carotenoid (fed with 0 and 88.6 mg/kg of feed, respectively). The same authors also observed a more intense pink colour in supplemented individuals. In a similar study with rainbow trout (*O. mykiss*), Zhang, et al. [132] observed the deposition of astaxanthin (8.0 and 8.6 mg/kg) in fish flesh following a feed supplementation with 100 mg/kg of this carotenoid. In opposition, this xanthophyll was not detected in the control group that did not receive this supplementation. On the other hand, Rahman, et al. [133] concluded that the supplementation of *O. mykiss* with astaxanthin at 50 mg/kg of feed increased its deposition in the muscle from 0.1 to 5.0 mg/kg, respectively. Yet, according to these authors, the supplementation with higher astaxanthin amounts did not lead to further improvements neither in carotenoid or astaxanthin concentration in rainbow trout muscle [133].

Within shrimp species, Dave, et al. [134] estimated a content of 44.52 mg/kg astaxanthin in Atlantic shrimp (*Pandalus borealis*). In this regard, other interesting sources of astaxanthin (with application in foods fortification, for example) including by-products like shrimp shells (that are usually wasted), can be considered. Chintong, et al. [135] found 28.9 mg/g in *Litopenaeus vannamei* shells, while Hu, et al. [136] determined levels of 50.32 mg/kg in *P. borealis* fresh shells and 239.96, 11.67, and 1.86 mg/kg in water boiled shells of *Procambarus clarkii*, *Portunus crab*, and Argentine red shrimp.

Meroterpenoids are defined as natural compounds that are partly derived from a terpenoid pathway. Regarding AD, the sargachromenol, sargaquinoic acid, and sargahydroquinoic acid are the most relevant substances. These meroterpenoids have the particularity to contain a quinone group in its terpenoid backbone, which is the reason why they are also known as plastoquinones. *S. sagamianum*, *S. serratifolium*, *S. sagamianum*, *S. micracanthum*, and *S. macrocarpum* are some of the seaweeds species where they were identified so far [137,138,139,140,141]. Choi, et al. [137] obtained 187 mg (0.04%) of pure sargaquinoic acid and 37 mg (0.01%) of sargachromenol in methanolic extracts obtained from 450 g of dried *S. sagamianum*. Even though these might seem rather low yields, it must not be forgotten that concentrations as low as 10.4 μM and 7.3 μM of sargaquinoic acid and sargachromenol, respectively, proved to elicit an anticholinesterase activity [137,139]. Kwon, et al. [142] produced ethanolic meroterpenoid-rich extracts from 1.5 kg of *S. serratifolium* with a yield of 3.0% (45.2 g), 0.5% (7.44 g), and 0.2% (2.27 g) in sargahydroquinoic acid, sargachromenol, and sargaquinoic, in the same order.

### 2.3. Sterols

Sterols comprise a type of lipids with an amphipathic nature that originate in isoprenoid biosynthesis. Structurally they are composed by a four-ring skeleton, a hydroxy group and a hydrocarbon side chain.

**Figure 6:**
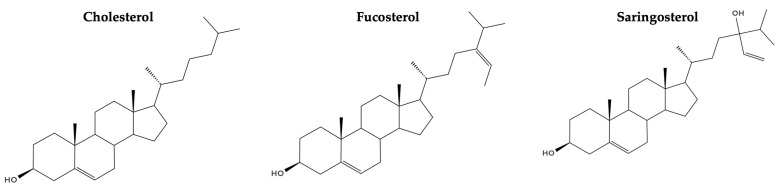
Molecular structures of cholesterol, fucosterol, and saringosterol.

Among animals, cholesterol is the most relevant, comprising approximately 20–25% of total lipids in plasma membranes [143]. Cholesterol (Figure 6) fulfils several biological functions, for example, as an essential structural and functional component of living cells where it is pivotal for the maintenance of membranes fluidity. It also serves as a precursor for the biosynthesis of a wide range of other molecules in organisms, including steroid hormones (like androgens, oestrogens, and glucocorticoids), bile acids and vitamin D [143].

Inside the cells, the biosynthesis of sterols relies in a complex process that takes place in the endoplasmic reticulum. During this process, multiple enzymatic reactions occur, leading to the formation of cholesterol in animals and phytosterols in plants. Sterols biosynthesized through the MVA pathway already described in Section 2.2, where acetyl-coenzyme A is sequentially converted into MVA, then in IPP and DMAPP, and finally in FPP. Two FPP molecules then combine to create squalene, a key intermediate in sterol biosynthesis. Then, squalene undergoes cyclization, being converted into lanosterol (in animals) or cycloartenol (in plants). From this point on, both lanosterol and cycloartenol are subjected to multiple modifications ultimately originating cholesterol (in animals) and phytosterols (in plants).

In mammalian cells, sterol levels are tightly regulated through a feedback-controlled system that oversees processes such as endogenous biosynthesis, dietary absorption, storage, breakdown into other sterols, and elimination. This regulation is crucial to maintaining an optimal balance of cholesterol and other sterols, which are essential for membrane stability and proper cellular function [144]. A key component in sterol biosynthesis regulation is the Sterol Regulatory Element-Binding Proteins (SREBPs). These proteins control the cascade of biosynthetic enzymes involved in the production of cholesterol, FA, TAG, and PL [145]. When intracellular cholesterol levels decrease, SREBP is activated and moves into the nucleus, where they upregulate the transcription of the genes involved in cholesterol synthesis and uptake to restore homeostasis. Contrariwise, when cholesterol levels are adequate, SREBPs remain inactive, thus reducing sterol production and absorption [146]. Another key element is the liver X receptors (LXRs) that act as “cholesterol sensors” and are responsible to maintain cholesterol homeostasis through transcriptional control of genes involved in sterol dynamics and transport, regulating its absorption, efflux, transport, and excretion [144,147]. Liver X receptors respond to sterol intermediates and metabolites, upregulating genes that enhance cholesterol efflux and metabolism while inhibiting excess cholesterol synthesis. Through these mechanisms, LXR are able to inhibit excessive cholesterol accumulation and maintain cholesterol homeostasis, thereby reducing the harmful effects associated with dysregulated cholesterol levels, particularly in the liver and brain. Since 3-hydroxy-3-methylglutaryl-coenzyme A reductase represents the rate-limiting enzyme in the hepatic de novo synthesis of cholesterol through the MVA pathway, it also plays a central role in cholesterol regulation [148,149].

Cholesterol homeostasis is essential for both cardiovascular and brain health, and its dysregulation has been associated with the progression of several chronic diseases, including cardiovascular disorders and neurodegenerative diseases such as AD. Increasing evidence suggests that vascular dysfunction and altered lipid metabolism may contribute to AD pathogenesis by influencing cerebral blood flow, neuroinflammation, Aβ peptides deposition, and neuronal function. In particular, abnormal cholesterol metabolism has been linked to Aβ peptides production and aggregation, tau pathology, and synaptic impairment. Therefore, maintaining balanced cholesterol homeostasis is considered important not only for cardiovascular protection but also for preserving cognitive function and brain health.

Many marine organisms, such as seaweeds, sponges, corals, and molluscs, are abundant in sterols. Among them, marine algae stand out as a particularly rich source of phytosterols, including fucosterol and saringosterol (Figure 6), with significant neuropharmacological benefits [4,150].

Particularly, fucosterol is pointed out as the most abundant sterol in marine algae [4], especially in brown seaweed which are recognized to produce higher amounts of this phytosterol than red and green seaweed [151].

As a result of the growing attention paid to fucosterol and saringosterol and their neuroactive potential, several marine seaweed species have been investigated. The study carried out by Martens, et al. [152] is a good example, where the authors analyzed the saringosterol and fucosterol contents in different European brown seaweed species (*Himanthalia elongata*, *S. muticum*, *Alaria esculenta*, *Ascophyllum nodosum*, *Fucus vesiculosus*, and *Saccharina latissima*). The sterols levels were determined both in the dry seaweed and in the respective extracts prepared in a 2:1 (*v*/*v*) chloroform/methanol mixture. Among the studied species *F. vesiculosus*, *H. elongate*, *S. fusiforme*, and *S. muticum* stood out with the highest contents of saringosterol (1.8–3.4 mg/100 g DW). Yet, the lipid extracts of *H. elongate* and *S. fusiforme* were the ones that provided higher amounts of this marine sterol (1.8 and 1.1 mM, respectively). Concerning fucosterol, *H. elongata*, *A. nodosum*, and *F. vesiculosus* were the most promising species (40.7–77.1 mg/100 g DW). In turn, the lipid extracts of *F. vesiculosus*, *F. muticum*, and *A. nodosum* were those that rendered the most relevant contents of fucosterol (12.2–13.0 mM). Yet higher yields were obtained when *S. fusiforme* was extracted by means of supercritical fluid extraction, where Martens, et al. [153] accounted 1.33 mM of saringosterol and 18.56 mM of fucosterol. In another study, Chen, et al. [154] reported a content of 30 mg/100 g DW of saringosterol and 59.5 mg/100 g DW of fucosterol obtained from the extraction of air-dried fronds of *S. fusiforme* with a 1:2 (*v*/*v*) dichloromethane/methanol mixture. Still the highest fucosterol levels were found to occur in leafy thalli from *Ecklonia stolonifera* collected in South Korea. The extract produced by Jung, et al. [155] with 500 g of lyophilized powder using methanol and subsequent purification with dichloromethane, ethyl acetate and n-butanol rendered a total of 390 mg of fucosterol (78 mg/100 g DW). Even higher contents were reported by Oh, et al. [156] that accounted 278 mg/100 g DW in the same seaweed. Fucosterol was also identified in *Panida australis* (6 mg/100 g DW), *S. horridum* (19.6 mg/100 g DW) [157,158].

## 3. Bioaccessibility/Bioavailability

The digestibility of marine lipids and the extent to which they are absorbed by the human body and become available to the target tissues—in the case of AD, the brain—is a key issue in connecting nutrition to health outcomes. In this context, defining bioaccessibility and bioavailability is of paramount importance and the associated concepts must be discussed. Whereas, according to Fernandéz-García, et al. [159], bioaccessibility encompasses all steps of digestive transformation up to the release from the food matrix into the intestinal lumen, absorption across the intestinal wall, and the pre-systemic metabolism (including the liver metabolism), for Paustenbach [160], bioaccessibility of a substance is defined as the fraction that is soluble in the gastrointestinal tract and is available for absorption. This second definition has been preferred in different studies [161,162,163]. Similarly, to Versantvoort, et al. [164], bioaccessibility is defined as the fraction of a compound, which is released from the food matrix during digestion in the tract and made available for intestinal absorption. On the other hand, bioavailability is defined as the fraction of the food compound ingested that is absorbed and utilized by the body for normal physiological functions [165], the brain being the key organ for the purposes of the current review.

Lipid bioaccessibility and, consequently, bioavailability depends on the chemical structure of the particular lipid form, physicochemical properties, food composition, and interaction with other food components [2]. Namely, it is known that calcium ions can bind to FFA and reduce the bioaccessibility/bioavailability of long-chain n-3 PUFA [166]. Hence, long-chain n-3 PUFA, either as FFA or bound in PL and TAG, may have variable bioaccessibility/bioavailability levels as a function of different food ingredients and meals.

### 3.1. Phospholipids

Within marine lipids, the bioaccessibility/bioavailability of PL has been a relevant subject of several research. In particular, it seems that PL are deemed more bioaccessible/bioavailable due to their amphiphilic biochemistry with stronger water-dispersibility and higher susceptibility to phospholipases in comparison to hydrolysis of TAG [32]. This entails a potentially higher degree of hydrolysis and suggests a higher availability for absorption across the intestinal wall.

However, it is enlightening to review the key facts of PL digestion. While digestion of TAG may start in the stomach, where they are hydrolyzed by lingual and gastric lipases, only very small amounts of PL are substrates for these enzymes [167]. Indeed, the large majority of the PL are hydrolyzed at the sn-2 position by the action of the pancreatic phospholipase A2 in the intestinal lumen, resulting in FFA and Lyso-PL crossing the enterocyte membrane [30].

The FA chain length and degree of unsaturation affect fat digestion, absorption, transport, and metabolism. It has been claimed that medium-chain FA are better absorbed than long-chain FA as a result of higher solubility in aqueous phase and related possibility of absorption in an albumin-bound form, which is transported directly to the liver [168]. Lyso-PL and a part of the FFA enter the bloodstream incorporated into the surface layer of chylomicrons after being re-esterified to PL. Various metabolic studies of orally administrated PL have shown that the cycle of deacylation/reacylation is a key metabolic aspect [169]. A small share of the PL will also be incorporated into very low-density lipoproteins (VLDLs) [30].

It has been reported that Lyso-PL can be an effective delivery form of DHA and other long-chain n-3 PUFA to the mammal brain, as studied in rats [60]. The investigation of the metabolism of EPA and DHA delivered either in PL (liposomes) or TAG form [170,171] has led to some relevant findings concerning n-3 PUFA bioavailability. In fact, it has been detected an increased n-3 PUFA bioavailability from PL in the lymph, when compared to n-3 PUFA from TAG [170,171]. In this regard, Ahmmed, et al. [2] concluded after a lengthy revision of several studies that PL n-3 PUFA are really more bioavailable and biologically active compared to n-3 PUFA in TAG form, but they also noted that PL n-3 PUFA have been ignored by the fish oil industry, resulting in a relative scarcity of PL products derived from marine sources when compared to TAG products.

Some key clinical and sub-clinical studies on bioaccessibility/bioavailability and applying different models to the estimation of these parameters should be mentioned. Regardless of the particular model, these various studies were mostly consistent with a higher bioaccessibility/bioavailability of PL n-3 PUFA with respect to n-3 PUFA in TAG or ethyl ester forms [172,173,174,175]. Concerning EPA and DHA and a human model, Bjørndal, et al. [176] found a substantially higher bioavailability of EPA and DHA in herring oil (containing 511 mg of total lipid, 30% of which are PL, with 56 mg EPA, 158 mg DHA, and 12 mg n-3 docosapentaenoic acid) than in a TAG-based supplement. Similar results were reported by Schuchardt, et al. [177]. Regarding the absorption rate of fish oil (mostly TAG) and krill oil (predominantly PL) in the brain and blood of an animal model, a direct comparison showed a lower amount of EPA in the blood of rats fed with fish oil than in rats receiving krill oil, 23.5% vs. 41.1%, thus pointing to a higher PL n-3 PUFA bioavailability [178]. In addition, algal oil, with high levels of PL, may be an alternative to krill oil. A systematic review [179] from intervention studies on the bioavailability of alternative vegetarian long-chain n-3 PUFA sources seems to strengthen this possibility. In particular, Lane, et al. [179] based their conclusions on studies showing that ingestion of algal oil enhanced DHA levels in the erythrocytes and plasma. It was concluded that though further research is warranted the algal oils may be an effective source of bioavailable long-chain n-3 PUFA [179].

Within PL themselves, n-3 PUFA bioaccessibility/bioavailability may also vary as a function of the polar head group in each specific PL [2]. The binding of long-chain n-3 PUFA in PL, SPL or PLG also matters [66]. These researchers were able to contrast the effect of EPA-rich PE and EPA-rich PLG PE on cognition improvement. They concluded that the latter exerted better effects on suppressing Aβ peptides generation [66], which may be connected to a higher bioavailability of PLG n-3 PUFA. Furthermore, the results involving DHA-rich PC, PE, and PS were interpreted by Ahmmed, et al. [2] as suggesting a variable long-chain n-3 PUFA bioavailability between these three types of PL. Therefore, there is still much research to be done regarding the bioaccessibility/bioavailability of PL.

### 3.2. Terpenoids

The bioaccessibility/bioavailability of terpenoids and other lipophilic compounds has not been as much studied as that of PL. Within this group of compounds, the carotenoids have special relevance and have received most attention [180]. Viera, et al. [180] have reported that the particular and diverse features of the marine matrix are influential for the carotenoid bioavailability, namely fucoxanthin. Data concerning this specific marine carotenoid come both from in vitro and in vivo bioaccessibility/bioavailability studies. In this regard, in vitro digestion protocols combined with differentiated Caco-2 cellular lines have been applied [180]. Under these experimental conditions, it was shown that although fucoxanthin was successfully accumulated by the Caco-2 cells, this marine carotenoid was poorly and less absorbed than other carotenoids, its absorption rate being similar to that of neoxanthin, a xanthophyll found in spinach [181]. Sugawara, et al. [181] also reported that Lyso-PC was able to enhance carotenoid uptake from mixed micelles by Caco-2 cells. Afterwards, Sugawara, et al. [182] observed that during fucoxanthin absorption by Caco-2 cells, the carotenoid was hydrolyzed to fucoxanthinol. Further studies showed that fucoxanthinol is converted into amarouciaxanthin A in human hepatoma HepG2 cells [183]. Moreover, in vivo studies in rodents have provided insight into the tissue distribution of fucoxanthin metabolites. In a study conducted by Yonekura et al. on rodents receiving 0.128 µmol of fucoxanthin/day for 14 days it was found that these metabolites (mostly amarouciaxanthin) accumulate mainly in the adipose tissue [184]. In addition, studies with human subjects have suggested the possibility of a lower affinity of the intestinal transporters for the less lipophilic epoxyxanthophylls, such as fucoxanthin, thus being the intestinal absorption of these carotenoids rather reduced in humans [183]. In fact, 6.1 mg (9.26 μmol) of fucoxanthin led to only 0.8 nmol/L of fucoxanthinol in plasma [183].

With respect to astaxanthin, another very relevant marine carotenoid, there have also been different applied methodologies. In particular, the level of astaxanthin absorption (originating from different sources) has been assessed by cell culture models established with Caco-2/TC7 cell lines [185] and in vivo experimental animal models [186]. Furthermore, intervention studies circumscribed to small human subjects have been carried out [187,188]. In these studies, different sources of astaxanthin (from wild and farmed salmon) and different intake patterns have been tested. Rüfer, et al. [187] have set up a randomized double-blind trial for assessing the bioaccessibility of astaxanthin from fish. An amount of 250 g of wild or farmed salmon with an astaxanthin content of 5 μg/g was given to the study subjects [187]. In this regard, it should be noted that, while the source of astaxanthin in the wild salmon diet is krill, farmed salmon eats feed supplemented with synthetic astaxanthin. This study revealed higher plasma astaxanthin concentrations in the participants consuming farmed salmon when compared to those eating wild salmon [187]. These authors observed a higher ratio of the (3S,3’S)-astaxanthin isomer to other isomers in plasma than in salmon flesh, which may hint at the possibility of modulating bioaccessibility through the pattern of isomerization. The conversion of astaxanthin into other compounds may also play a role, with four metabolites being mentioned in this context [180]: 3-hydroxy-4-oxo-β-ionol, 3-hydroxy-4-oxo-β-ionone and their corresponding reduced forms [189,190]. Within these biochemical issues, it has been claimed that the absorption through the intestinal epithelium presupposes the hydrolysis of xanthophyll esters in the intestine [180]. This has been related to the detection of free forms in plasma after consumption of food rich in xanthophyll esters [191,192]. Hence, the issue of the astaxanthin bioaccessibility/bioavailability has several layers of biochemical complexity.

In general, it can be summarized that the factors affecting carotenoid bioaccessibility and bioavailability are still not fully understood [180,193]. These necessarily comprise the carotenoid release from the food matrix, the incorporation into mixed bile micelles, the carotenoid transfer from micelles to the intestinal mucosa for passive or facilitated absorption, and the sequestration into chylomicrons. Besides dietary factors, carotenoid bioaccessibility/bioavailability may also depend on the specific carotenoid structure, being, for instance, polar carotenoids preferably incorporated into mixed micelles and more bioavailable [193].

### 3.3. Sterols

Algal sterols, such as fucosterol or saringosterol, are another group of marine bioactive substances whose fate after digestion has also been researched. However, these studies are very scarce and represent an area with substantial knowledge gaps. The main locus of sterol absorption is the intestine. However, they are usually present only at low plasma levels in humans due to a reduced net absorption rate by the small intestine [194]. A seminal study by Vahouny, et al. [195] showed that fucosterol—alongside sitosterol and stigmasterol—was poorly absorbed to the extent of only 3–4% of the administered dose of 50 mg. The cholesterol absorption used as a comparing reference reached approximately 42% of the administered dose [195]. Moreover, whereas administration of sitosterol and stigmasterol led to an effective inhibition of cholesterol absorption, fucosterol did not manifest any inhibitory effect. Accordingly, Vahouny, et al. [195] concluded that the process(es) underlying intestinal discrimination of sterols as expressed by the absorption rates may differ from the mechanism(s) explaining cholesterol uptake interference. Recent research as revised by Cedó [196] has confirmed this by showing that phytosterols do not need to be absorbed to be highly effective. Indeed, they are able to compete with dietary and biliary cholesterol for micellar solubilization, impairing intestinal cholesterol absorption [196]. Phytosterols also have a role in the transintestinal cholesterol excretion pathway by promoting cholesterol flow from the blood to enterocytes and into the intestinal lumen, in the conversion of bile acids into secondary bile acids, and in lowering the bile acid hydrophobic/hydrophilic ratio, thus limiting intestinal cholesterol absorption [196].

For saringosterol, a recent study by Abdelrheem, et al. [197] used molecular structural data to estimate that this marine sterol may have a high bioavailability. Indeed, its topological polar surface area does not surpass 160 Å and it does not violate any of the Lipinski’s rules of five—no more than 5 H bond donors; no more than 10 H bond acceptors; a molecular mass lower than 500 Da; and an octanol-water partition coefficient not exceeding 5 [197]. Nonetheless, such promising inferences must still be supported by substantive experimental data. All the observed absorption variability between compounds may be relatable to intestinal lumen selectivity processes and to a phenomenon of return, occurring by an adenosine triphopsphate (ATP) Binding Cassette-transporter–mediated process, which may also explain the dependence of the absorption efficiency on the structure of both sterol nucleus and side chain [198].

Because dietary sterols have a similar structure to cholesterol and follow the same absorption pathway, they interfere with intestinal cholesterol uptake, ultimately helping to maintain cholesterol balance within the body [4].

Yet, on the contrary to cholesterol, fucosterol and saringosterol have already been identified in the brain, suggesting that they are able to cross the blood–brain barrier (BBB) and accumulate in brain parenchyma [4,152,199]. Even though the route of entry of this phytosterol in the central nervous system is not entirely understood, it is hypothesized that it may occur via transcellular diffusion, similarly to what is known for 24(S)-Hydroxycholesterol, which is an oxidized sterol as well [199]. Indeed, Martens, et al. [200] observed that the administration of 24(S)-saringosterol led to a significant increase in this phytosterol both in the serum and the brain, while fucosterol in circulation and the brain was diminished. These authors present several possible explanations for the reported trends: 1) competition for incorporation in micelles and the subsequent intestinal absorption, 2) an inhibitory effect of 24(S)-saringosterol on intestinal sterol absorption, or even 3) an enhanced effect of 24(S)-saringosterol on sterol excretion via LXR activation.

The low bioaccessibility may be partially overcome with strategies that modulate sterol solubility, for instance, by increasing fucosterol significantly on its complexation with maltosyl-β-cyclodextrin [201]. It would be very important to find ways to enhance bioaccessibility/bioavailability and to have a deeper knowledge of the interaction between molecular structure and absorption as well as biological activity of algal sterols [11].

## 4. Metabolism of Marine Lipids

The metabolism of marine lipids comprises all biochemical transformations that occur from the time that these molecules contact with the human digestive system (see also previous section) to either their final decomposition for their utilization as an energy source or their biosynthetic reaction products, which may constitute structural building blocks in different tissues (namely in neuron cell membranes) or signalling and regulatory compounds in the most diverse organs [170,180,194]. It should also be noted that lipid metabolism is a complex process involving various enzymes, hormones, genetic factors, and their interactions with each other [202]. The liver is crucial for the regulation of lipid metabolism and controlling many processes such as FA anabolism, FA catabolism, and cholesterol uptake [203,204].

A central metabolic problem of lipids in the human organism is the occurrence of hyperlipidaemia, which is characterized by high levels of low-density lipoprotein (LDL) and low levels of high-density lipoprotein (HDL) [49]. In this regard, marine lipids, such as long-chain n-3 PUFA, can effectively prevent the occurrence of hyperlipidaemia. Moreover, such bioactive marine lipids are natural ligands for various nuclear receptors that regulate gene expression, encompassing the peroxisome proliferator-activated receptor, hepatocyte nuclear factor, LXR, and retinoid X receptor. They also modify the expression of some transcription factors, namely SREBP and carbohydrate response element binding protein [205,206].

Another biochemical system sensitive to n-3 PUFA is the endocannabinoid system including two G protein-coupled receptors (cannabinoid receptor type 1 and type 2), endogenous ligands (anandamide, 2-arachidonoyl glycerol, nolandin ether) and enzymes with the ability to decompose endocannabinoids [207,208]. It should be remarked that anandamide is chemically N-arachidonoylethanolamine and nolandin ether is 2-arachidonylglycerylether. Hence, all these endogenous ligands are related to the n-6 PUFA arachidonic acid (20:4 n-6). Accordingly, dietary FA may affect the metabolic system linked to endocannabinoid action by modulating arachidonic acid concentration in tissue PL. In particular, the marine n-3 PUFA could drive a reduction in the amount of arachidonic acid substrate available in membrane PL for the synthesis of the endogenous ligands, thereby down-regulating an overactive endocannabinoid system [49]. Furthermore, the long-chain n-3 PUFA can be used as endocannabinoid substrates for the generation of docosahexaenoylethanolamide and eicosapentaenoylethanolamide, which are able to bind to cannabinoid receptor type 1 and type 2 and counterbalance the action of anandamide and other 20:4 n-6 derivatives [49,208,209].

Furthermore, it is well known that one of the main metabolic fates of DHA in the brain is to form a protective docosanoid, known as neuroprotectin D1 (NPD1), which has a potent anti-inflammatory and neuroprotective activity [210]. This DHA derivative decreases Aβ 42 peptides release from ageing brain cells and is drastically reduced in AD brains [211]. This metabolic connection between DHA and NPD1 is part of the larger issue of signalolipidomics [212]. Neuroprotectin D1 is considered a DHA-derived stereoselective mediator whose synthesis agonists include neurotrophins and oxidative stress. This neuroprotectin leads to strong anti-inflammatory actions as well as prohomeostatic bioactivity, being also anti-angiogenic and inducer of cell survival [212,213].

All these metabolic aspects of the presence of different bioactive lipids in the body may affect the onset and the progression of dementia and, in particular, AD. The challenge lies in articulating the relationship between particular lipid molecules and neurodegenerative outcomes through a dense and imbricated net of metabolic pathways and interactions that necessarily involve inflammatory processes and various mutually dependent signalling systems. In Table 2 is presented a comprehensive summary of the compounds addressed and their respective mechanism of action and/or reported health impact against AD pathology.

### 4.1. Phospholipids

It is fundamental to achieve a deep understanding of PL metabolism in the human organism in order to establish links between the presence of specific molecules in seafood and measurable health outcomes. This requires investigation of biochemical processes occurring in the gastrointestinal tract, liver, blood, and target tissues. Such studies present multiple challenges that can be addressed using both in vitro and in vivo approaches. In particular, in vivo investigations may involve advanced and costly imaging techniques, especially for brain-related studies in humans, such as Positron Emission Tomography which is primarily applied in clinical research settings and selected translational studies, but can also be used preclinical research settings [29,256].

The metabolism of PL is clearly different from that of TAG, being reported that there is a higher n-3 PUFA bioavailability from PL in lymph [35,170]. Of course, this constitutes a precious advantage of PL over TAG. Regarding this subject, it is worth noting that n-3 PUFA, such as EPA and DHA, are able to inhibit the metabolism of arachidonic acid by cyclooxygenase-1 (COX-1), thus operating as anti-inflammatory agents [257]. Accordingly, n-3 PUFA PL may more strongly inhibit inflammatory metabolic processes than n-3 PUFA TAG. In fact, it is known that after digestion and lipid absorption, the TAG-rich particles of the chylomicron are decomposed, enabling PL such as PC to be taken up by the HDL fraction [30]. This process occurs relatively rapidly, within 5 to 6 h after PL ingestion [258]. Afterwards, in this form, PL can be transferred into cells in different tissues and locations (e.g., muscle, lung, etc.). Accordingly, whereas TAGs are more intimately connected to energy supply in the organism and readily decomposed—hydrolyzed by lipoprotein lipase—to provide energy as demonstrated by experiences with parenteral administration of fat emulsions, PL may be transferred to cell membranes and, as a function of the type of their FA, these lipid molecules can modulate the synthesis of eicosanoids and docosanoids [212,259].

In this context, the PL transfer protein has a very important role in PL metabolism [260]. PL transfer protein is a glycoprotein with 476 residues, which facilitates the transfer of a wide spectrum of lipid molecules, including DAG, PA, SM, phosphatidylglycerol (PG), CER, PE, and PC [261]. There are two forms of functionally defined PL transfer protein in human plasma—a so called active form that is able to transfer PC from PL vesicles to HDL and an alternative form that lacks this ability [262]. This latter form may have other relevant lipid transfer-independent functions on the cell surface, such as signal transduction activation [260]. In this regard, it must be referred that there is evidence suggesting a link between HDL and related proteins in plasma and AD [263]. More recently, Whiley, et al. [264] found out that PC metabolism is altered in the case of AD patients, but the exact mechanism is not understood. However, there are several related associations; for instance, an investigation identified a 16:0/16:0 PC as a predictive marker of AD development in individuals showing mild cognitive impairment (MCI) [265]. Confirmatory investigations into the background biochemistry by Whiley, et al. [264] revealed no significant change in total choline-containing compounds or total plasma omega FA, thus reinforcing the possibility that specific PC molecules are critical in AD pathology. In particular, three PC molecules were found to be significantly lower in AD patients, 16:0/EPA PC, 16:0/DHA PC, and 18:0/DHA PC, by Whiley, et al. [264]. The presence of EPA and DHA in these PC molecules emphasizes the importance of marine long-chain n-3 PUFA to brain health [209].

Moreover, studies on animals [44] have shown that EPA-PC and DHA-PC could significantly decrease Aβ peptides levels in CHO-APP/PS1 cells and SAMP8 mice by inhibiting APP, presenilin 1 (PS1), and beta-site amyloid precursor protein cleaving enzyme (BACE1) expression. Moreover, besides other effects, both EPA-PC and DHA-PC significantly suppressed apoptosis through changes concerning the B-cell lymphoma 2 regulator proteins and lower expression of pro-apoptosis factors [44]. On the other hand, only DHA-PC could ameliorate the expression of neurotrophic factors, including brain-derived neurotrophic factor (BDNF), synaptophysin, and growth-associated protein 43.

Another specific and relevant group of PL is formed by the phosphorylated derivatives of PI, also called phosphoinositides, which, besides other functions, are important in lipid and cell signalling as well as membrane trafficking [37]. Seven interconvertible species of phosphoinositides were usually believed to be the key actors in all these functions through their ability to recruit a specific set of cognate effector proteins. However, it seems the phosphoinositides molecular diversity is much larger, given the variety of acyl chains carried by phosphoinositides [266], and their interconversion is probably achieved through kinase–phosphatase enzyme complexes that coordinate their activities without affecting bulk substrate population [37,267]. In the particular case of scyllo-inositol, a naturally occurring isomer of inositol found in the human brain, recent research [256] has found evidence that *scyllo*-inositol can be incorporated into PI in mammalian cells, suggesting a broader, previously underappreciated role in cell metabolism. It has been proposed that it works by interacting with Aβ peptides in the prevention of the formation of toxic plaque aggregates in the brain [215,268]. Effects on plaque counts were seen with intakes exceeding 1 mg/(kg body weight.day) in a mouse model [268]. All this means that further research on the metabolic fate of these PL is required, being potentially insightful for a better understanding of the connections between phosphoinositides, diet, and brain health.

Within the group of PL, cardiolipin (CL), 1,3-bis(sn-3′-phosphatidyl)-sn-glycerol, is a very relevant component of the inner mitochondrial membrane, where it is crucial for the optimal function of many enzymes that are associated with the mitochondrial energy metabolism [38,269]. In mammals, CL is synthesized via the cytidine-5′-diphosphate-1,2-diacylglycerol pathway, which involves glycerol-3-phosphate, PA, and PG as precursors [270]. Once PG is synthesized, it is rapidly converted to nascent CL by the enzyme CL synthase. Though this enzyme does not have a strict acyl chain specificity, as demonstrated in studies carried out in different organisms [271,272], the CL occurring in membranes contains specific acyl chains that are decisive in its function. This apparent contradiction may be related to the remodelling process of nascent CL that leads to the incorporation of specific acyl groups into CL before its membrane integration [38]. It should be noted that CL, as well as its remodelling, is critical for the development and health, playing a significant role in mitochondrial-mediated apoptosis [38]. Namely, cells may undergo mitochondria-dependent apoptosis, which requires CL for its induction [273]. It has been shown in the model organism *Caenorhabditis elegans* that CL supplementation (100 µg/mL) has a restoring effect on mitochondria and may contribute to the homeostasis of the central nervous system [216,274].

Though a minority group within PL, PLG may have a key role in the functioning of the human organism [30] in particular, given their lipid signalling bioactivities. A paradigmatic example is the platelet-activating factor, which is also known as 1-*O*-alkyl-2-acetyl-sn-glyceryl-3-phosphorylcholine [275], a PLG. This is a potent inflammatory mediator associated with the innate immunity and chronic inflammatory diseases [276,277]. On the other hand, ethanolamine PLG are considered to be neuroprotective lipid molecules [51], their levels being reduced in AD brains. Sejimo, et al. [51] proved that these PL can substantially attenuate the microglial expression of protein kinase C delta (PKCδ) in a specific neuroinflammation model and in mice brains. The existence of a possible link between PKCδ activation and neuroinflammation should be highlighted. Sejimo, et al. [51] also observed an increase in PKCδ in human AD brains analyzed post-mortem. Moreover, it was claimed in this recent study that PLG may inhibit the p38 mitogen-activated protein kinase (MAPK) and c-JUN N-terminal kinase (JNK) protein expression, which is meaningful given the involvement of the p38 MAPK and JNK systems in the expressional regulation of PKCδ in microglial cells [51]. The specific mechanism of action for the ethanolamine PLG has been illuminated by different experimental works [217,278] and it has been pointing to an upregulation of the tropomyosin receptor kinase B/extracellular signal-regulated kinases/cyclic AMP-dependent response element-binding protein signalling pathway. One of these studies [217] has found out that EPA-enriched ethanolamine PLG (up to a purity of 93.4%) is more prominent than EPA-enriched PE in upregulating this pathway, being also relevant its effects on inhibition of oxidative stress and suppression of apoptosis. Effects were detectable with a 10 µg/mL concentration of ethanolamine PLG [217]. The recognition of these effects has already led to experimental exploration of synthetic PLG precursors [279].

The understanding of the metabolic fate of PL in the human organism and, in particular, in the brain also requires a deeper knowledge of the genetic variability between individuals. A thorough knowledge of all metabolic mechanisms is very important to understand why specific PL molecules have a positive health impact—namely, on the brain—and others do not. It has been posited that whereas free DHA is transported across the outer membrane leaflet of BBB through passive diffusion, DHA-Lyso-PC is transported across the inner membrane leaflet of the BBB through the key facilitator superfamily domain-containing protein 2A [209,280]. In carriers of the APOE ε4, brain transport of free DHA, but not of DHA-Lyso-PC, may be impaired as a result of alterations in the outer membrane leaflet [280]. Patrick [280] claimed that the dietary form affects this asymmetry; DHA in ethyl ester or TAG (as in some supplements) or even as DHA-PL with DHA in the sn-2 position generate both FFA and re-esterified DHA-Lyso-PL, thus diminishing the possibilities of crossing the BBB. Moreover, contrary to Thies, et al. [60], it was defended as an advantage for DHA at the sn-1 position of PL [280]. In contrast to DHA at the sn-2 position, DHA at the sn-1 position would escape the action of pancreatic phospholipase A2, thus allowing all of the DHA to be retained in PL form and augmenting the probability of DHA-Lyso-PC generation [34]. Sustaining this view, it was reported that DHA in the sn-1 position of PL accumulates in the HDL fraction by more than twofold [34]. Hence, different metabolic routes and fates may lead to different levels of bioactivity in the target tissues and help to explain different health outcomes.

The metabolic phenomena underlying AD remain still elusive, given the insufficient understanding of the multiple systems operating in the nerve cells. The cholinergic and dopaminergic systems belong to those more extensively studied in the attempt to identify causes for neurodegenerative diseases. The cholinergic system is formed by the organized nerve cells that use the neurotransmitter acetylcholine (ACh) in the transduction of action potentials. Acetylcholine has choline as precursor and metabolite, which participates in several relevant neurochemical processes, has a role in single-carbon metabolism, and is a component of PC [281]. On the basis of decreased cholinergic neurotransmission in brain disorders characterized by cognitive impairment, cholinergic precursor loading therapy with choline-containing PL was tried as medical treatment in ameliorating AD symptoms [281]. This therapy was abandoned due to the negative clinical results obtained with choline or lecithin, which also comprises PC. Such compounds were probably not effective at enhancing brain levels of ACh [282]. Other PL involved in choline biosynthetic pathways, such as citicoline—an intermediate in the generation of PC from choline; choline alphoscerate—L-alpha glycerylphosphorylcholine, a choline compound found in the brain; and, also, PS, effectively enhanced ACh bioavailability or release in in vivo models and were able to ameliorate cognitive function in AD patients [282,283]. Anyway, PC and Lyso-PC may, at least, have diagnostic value. Klavins, et al. [284] concluded that the ratio of PC to Lyso-PC in plasma differentiates healthy individuals from patients with AD and MCI.

The study of the dopaminergic system is also relevant and shows connections and sensitivity to PL that exist in seafood. In fact, the metabolic routes involving the regulation of the dopamine system are deemed important for neurodegenerative diseases. In this regard, Wang, et al. [218] concluded that PL containing DHA were able to increase the number of dopaminergic neurons in 1-methyl-4-phenyl-1,2,3,6-tetrahydropyridine-induced mice, this effect being stronger with DHA-PS than with DHA-PC. The mechanism underlying this positive effect may be related to the action of these molecules in the inhibition of apoptosis via mitochondria-mediated pathway and MAPK pathway [218]. The special efficacy of DHA-PS is possibly a result of PS being the major acidic PL, accounting for 13–15% of the PL in the human cerebral cortex [285]. In the plasma membrane, PS is found exclusively in the cytoplasmic leaflet where it constitutes part of the protein docking sites, which are required for the activation of various key signalling pathways [286]. These comprise the protein kinases B and C as well as other kinases signalling pathways, which are known to stimulate neuronal survival, neurite growth, and synaptogenesis [287,288,289].

The balance between different neurotransmitters systems such as ACh, dopamine, noradrenaline, gamma-aminobutyric acid, serotonin, and glutamate is crucial for brain function [290] and is disturbed in AD patients. In any case, the whole metabolism of PL is still not fully investigated and many questions remain unanswered. In order to understand the role of PL after their ingestion and the way they articulate with the biochemistry of the human metabolism, more research is warranted. Hence, many of the PL effects on the human organism remain undiscovered, awaiting results from relevant clinical trials that are still underway [29].

### 4.2. Terpenoids

The biological activity of terpenoids in the pathophysiology of neurodegenerative disorders like AD comprise a wide array of mechanisms with special emphasis: 1) inhibition of inflammatory processes (through the reduction in cytokine secretion and inhibition of the Nuclear factor-κB (NF-κB) pathway), 2) antioxidant effects (via prevention in DNA damage or the inhibition of the production of reactive oxygen species (ROS)), 3) anticholinesterase activity (by inhibiting the activity of AChE and BChE enzymes), 4) anti-amyloidogenic action and Aβ peptides clearance (namely through the inhibition of BACE1) [103,239,252,291,292,293].

The study conducted by Machado and his colleagues [77] highlighted a positive effect of the red macroalgae *O. secundiramea* extracts (produced with dichloromethane:methanol, 2:1 *v*/*v*) over the AChE inhibition. The authors related this biological activity with the fraction containing halogenated monoterpenes, with both bromide and chlorinated species. This effect has been attributed to a reversible competitive inhibition of AChE [294]. More importantly, *O. secundiramea* extract did not exhibit cytotoxic or mutagenic effects [295].

Shanmuganathan, et al. [84], [227,296] have carried out multiple studies focused on acetone extracts of *P. gymnospora* and its active compound α-bisabolol. The α-bisabolol found in the algal extracts exhibited an anti-cholinesterase activity (at a concentration for 50% inhibition [IC_50_] value of <10 mg/mL) and the ability to prevent the oligomers formation and disaggregation of mature fibrils [84]. These results, initially measured by enzymatic assays, were later corroborated by in vitro and in vivo studies. Treatment with α-bisabolol (5 μg/mL) was able to prevent and reduce the aggregation propensity of Aβ peptides oligomers, as well as the disaggregation of mature fibrils [296]. Furthermore, a rescuing effect against Aβ peptides-induced neurotoxicity and a neuroprotective activity against Aβ25-35-induced apoptosis was also reported both for PC12 and Neuro2a cells [227,296]. The co-treatment with *P. gymnospora* acetone extracts (25 μg/mL) and the α-bisabolol (5 μg/mL) were also ascribed an important antioxidant activity against ROS and reactive nitrogen species, contributing to revert and prevent the Aβ-induced oxidation processes in Neuro2a cells. In addition, the in vivo studies using transgenic *Caenorhabditis elegans* strains of AD confirmed the neuroprotective activity against Aβ25-35 peptides-induced processes and the ability to prevent the amyloidogenesis of both the *P. gymnospora* acetone extracts (25 μg/mL) and α-bisabolol (25 μg/mL) [227].

The interest in aplysistatin isolated from *L. snackeyi* (Weber-van Bosse) as a potential compound to treat neurodegenerative diseases is related to its anti-inflammatory effect [80,102], since neuroinflammation is an important pathogenic factor of AD [297]. According to Vairappan, et al. [80], aplysistatin (at 20 μg/mL) inhibited the NO and prostaglandin-E2 (PGE2) production, suppressed inducible nitric oxide synthase (iNOS) and COX-2 expression in LPS-stimulated RAW 264.7 cells.

Caulerpenyne is considered to be a highly bioactive molecule that has been linked to anticancer activity, cell grown inhibition, and neurotoxic activity [67]. Cengiz, et al. [229] studied methanolic extracts of *C. prolifera* and observed that these algae extracts (at a final concentration of 48 mg/mL) inhibited at nearly 80% of the soybean lipoxygenases (LOX). According to these authors, the found activity was due to the presence of caulerpenyne. These LOX enzymes promote the oxidation of unsaturated FA, catalyzing the addition of oxygen of several FA like linolenic and arachidonic acids, as well as DHA and other PUFA originating new biologically active compounds. The subsequent in vitro enzymatic assays revealed an IC_50_ value of 5.1 µM for the inhibition of soybean LOX [229]. This is a quite relevant biological activity considering that, in mammalians, LOX enzymes participate in the production of mediators of several disorders like bronchial asthma, allergic rhinitis, inflammatory bowel, skin diseases, rheumatoid arthritis, cancer, osteoporosis, and cardiovascular diseases [229]. In turn, these will then significantly impact the course of neurodegenerative diseases such as AD [298]. Particularly the 5-LOX and the 12/15-LOX seem to be the most important isoforms concerning neuroinflammation/neurodegeneration [298].

Multiple biological activities have also been described for zonarol, including its anti-fungic and anti-inflammatory properties, and more recently, its neuroprotective effect [299]. Shimizu, et al. [86] found that treatment of HT22 hippocampal neuronal cells with zonarol extracted from *Dictyopteris undulata* led to dose-dependent protection from the cell death caused by glutamate-induced oxidative stress. Contents between 1 and 10 µM were tested. The neuroprotective effect was also recorded for cerebrocortical neurons. The experiments developed by this same team led the authors to conclude that this effect occurred via the activation of the Nuclear factor (erythroid-derived 2)-like 2/antioxidant-responsive element (Nrf2/ARE) pathway. The activation of this pathway consists of an intrinsic mechanism in order to protect the cells from oxidative stress-induced cell death [300]. The mechanism behind this activation relies on the chemical properties of zonarol *para*-hydroquinone ring, making it a pro-electrophilic compound that, through oxidation, can be converted to a quinone-type electrophile and activate the Nrf2/ARE pathway [86,301].

In 2003, Ryu, et al. [87] isolated two farnesyl acetone derivatives, (5e,10z)-6,10,14-trimethylpentadeca-5,10-dien-2,12-dione (dihydromonofarnesylacetone, C_18_H_30_O_2_) and (5E,9E,13E)-6,10,14-trimethylpentadeca-5,9,13-trien-2,12-dione (monooxofarnesylacetone, C_18_H_28_O_2_), from the Korean brown alga *S. sagamianum*, which were demonstrated to possess moderate AChE and BChE inhibitory activities. Interestingly, these farnesyl acetone derivatives share a common skeleton with other potent BChE-inhibitory plastoquinone derivatives, sargaquinoic acid, sargachromenol and sargahydroquinoic acid (that will be discussed later), that were also isolated in *S. sagamianum* and *S. serratifolium* [87,139]. According to Barbosa, et al. [102], these results might suggest that molecules’ scaffold may be a relevant feature in the modulation of the cholinesterase inhibition.

Vitamin A (retinol) and its metabolites have been linked to numerous physiological brain functions, including the control of neuronal differentiation during development and modulation of neuronal plasticity and neurogenesis in the adult hippocampus [302].

Goncalves and her colleagues [303] reported that the Aβ peptides downregulate the retinoic acid synthesis. On the other hand, Corcoran, et al. [304] related the deficiency in vitamin A with the augmented Aβ peptides production, while Zeng, et al. [305] registered an increase in the neuritic plaque formation with lower vitamin A levels. On the contrary, the treatment of AD model mice with vitamin A showed lower Aβ peptides deposition and tau phosphorylation as well as lower neuronal degeneration [230]. Takasaki, et al. [306] also observed that retinoic acid decreased cellular toxicity by inhibition of Aβ42 peptides aggregation in a dose-dependent manner. These authors suggested that such effect could be attributed to the interaction of retinoic acid with the peptides’ C-terminal portion [306]. According to Grimm, et al. [103], vitamin A and its derivatives activity concerning the Aβ peptides reduction seem to be related with their retinoid-dependent transcriptional regulation of AD-relevant genes like those encoding for APP, BACE1, PS1, and PS2.

Another study conducted by Watamura, et al. [231] highlighted the presence of fewer and smaller tau aggregates in the brain tissue of 33 × Tg transgenic mice treated with all-*trans*-retinoic acid. According to these authors, this effect is probably due to the downregulation of the cyclin-dependent kinase 5 and the glycogen synthase kinase 3β and the activation of astrocytes and microglia in the hippocampus.

Since vitamin A is primarily stored in the liver, Li, et al. [307] hypothesized that AD could alter its hepatic metabolism. To investigate this, they studied two types of mice: control (C57BL/6J) and AD model (APP/PS1). Each group was further divided and fed either a standard diet containing 4000 IU/kg of vitamin A or the standard diet with low vitamin A containing 400 IU/kg. Although plasma vitamin A levels remained consistent across all groups, liver vitamin A levels were significantly lower in mice on the low-vitamin A diet. Interestingly, Li, et al. [307] also found elevated hepatic levels of Retinol-Binding Protein 4, which transports retinol from the liver to peripheral tissues via the bloodstream. These findings suggest that blood retinol concentrations are tightly regulated and may not accurately reflect liver vitamin A reserves, until those reserves are critically low.

Vitamin D is a triterpenoid that has been consistently related with neuronal health and a normal cognitive function. Among other aspects, the fact that both vitamin D receptor (VDR) and the 1α-hydroxylase (enzyme responsible for the conversion of vitamin D in calcitriol) are present within multiple brain tissues [308], while low levels of VDR mRNA were determined in the brains of AD patient’s post-mortem [309], reveals the paramount importance of vitamin D for the brain physiological functioning. A vitamin D deficiency is closely associated with a heightened risk of developing cognitive impairment and with the progression of AD [310]. Furthermore, higher levels of vitamin D-binding protein and lower amounts of 25-hydroxyvitamin D in the cerebrospinal fluid have been observed in AD patients [311].

So far, numerous studies have already reported that vitamin D intervenes in diverse pathophysiological aspects of AD. For example, its anti-inflammatory effect (through the inhibition, for example, of tumour necrosis factor-alpha (TNF-α) and interleukin (IL)-6 production), antioxidant action, control of calcium homeostasis, inhibition of tau hyper phosphorylation, and the reduction in the amyloid burden (achieved at the expense of both reduction in Aβ peptides production and increasing amyloid clearance) are some of the mechanisms of AD pathogenesis where vitamin D participates [232,233,292,309,310,311,312]. Hypotheses proposed to explain the pathways by which vitamin D modulates the Aβ peptides load include: 1) an increased brain-to-blood efflux transport of the peptides at the blood–brain barrier and a stimulation of microglial Aβ peptides phagocytosis and 2) increase in neprilysin enzymes (Aβ-degrading enzymes) and a reduction in BACE1 expression [232,313]. These findings seem to agree with Grimm et al. [314] observations, that reported an increased BACE1 activity combined with a reduced neprilysin enzymes activity in the brain tissue of vitamin D-deficient mice. In addition, the downregulation of the VDR mRNA, found in individuals with AD [315], seems to be relevant, since VDR takes part in the reduction in Aβ peptides accumulated in neuronal tissues [234], and its overexpression might suppress the APP [316].

In what concerns AD, the pharmacological potential carotenoids, and some xanthophylls in particular, have been related with an important anti-amyloidogenic and aggregation inhibition activity, anticholinesterase activity, as well as their antioxidant and anti-inflammatory effect [239]. This is the case of fucoxanthin which has been recognized to act over multiple AD biomarkers [317]. Based on Jung, et al.’s [237] findings, the fucoxanthin extracted from *U. pinnatifida* was able to tightly binding of the active site of BACE1. According to the results presented, the two hydroxyl groups present in fucoxanthin interact with two specific amino acid residues of BACE1 enzyme, thus inhibiting its activity and the production of Aβ peptides. In turn, fucoxanthin also seems to prevent the toxicity induced by Aβ peptides oligomer and thus avoid neuronal loss and oxidative stress [115]. More recently, Alghazwi, et al. [242] looked up for the fucoxanthin and astaxanthin neuroprotective potential, and found that both xanthophylls inhibit Aβ peptides aggregation and the subsequent cytotoxicity and apoptosis. The same authors also reported fucoxanthin and astaxanthin protective effects against ROS. Overall, although both carotenoids showed beneficial effects, fucoxanthin proved to be more effective at lower concentrations. Xiang, et al. [116] demonstrated that the fucoxanthin anti-aggregation activity is probably due to its hydrophobic interaction with the Aβ peptides, which makes difficult the Aβ 1-42 peptides fibrils and oligomers formation.

The investigations carried out by Kawee-ai, et al. [118] and Lin, et al. [238] have focused on the fucoxanthin anticholinesterase activity. Despite former authors having evaluated the inhibition of both AChE and BChE, only BChE was inhibited by fucoxanthin (IC_50_ value of 1.97 mM). Lin et al. [238] instead, have just studied the AChE, where they have determined an inhibition with an IC_50_ value of 81.2 µM. These authors suggest that the fucoxanthin is likely to interact with the peripheral anionic site within AChE.

When compared to anticholinesterase activity, for example, the antioxidant and the anti-inflammatory actions may be less specific for the AD. Still, they undoubtedly contribute to an important neuroprotective action by attenuating the neuronal cell damage, inhibiting cell apoptosis, and preventing DNA damage, among other possible protective actions [102]. A neuroprotective potential, related with antioxidant activity, has been repeatedly identified in fucoxanthin and fucoxanthinol extracted from multiple brown seaweeds (*H. elongata*, *S. horneri*, *U. pinnatifida*, *S. siliquastrum* [102,239,252]. Apparently, the antioxidant potential of fucoxanthin and fucoxanthinol is due to their allenic bond as well as the acetyl group in fucoxanthin [318].

Also noteworthy is fucoxanthin’s ability to attenuate inflammation processes by modulating the secretion of proinflammatory mediators that intervene in neurodegenerative pathologies like AD, including iNOS, COX-2, and the phosphorylation of MAPK, but also TNF-α, IL-6, IL-1β, and PGE2 [239].

The relevance of fucoxanthin’s potential for the treatment of neurodegenerative pathogenesis like AD, gains a special interest since it has been pointed as safe for consumption, with no visible toxicological effects [319].

Besides fucoxanthin, astaxanthin is another xanthophyll highlighted for its significant neuroprotective action mainly due to anti-inflammatory action and potent antioxidant activity that is able to counteract the neuro-inflammatory processes and the oxidative stress that are at the base of the AD. In their study, Rahman, et al. [243] described that the treatment of AD-induced Wistar rats with astaxanthin had an anti-amyloidogenic effect on the hippocampus, along with the reduction in TNF-α level, the AChE activity, and the oxidative stress.

Astaxanthin’s ability to suppress the ROS resulted in lower SH-SY5Y and PC12 cells apoptosis, while its anti-inflammatory activity contributed to attenuate the expression of iNOS and COX-2, as well as the IL-6 and the MAPK signalling pathway [132,244,245]. Alghazwi, et al. [242], observed that astaxanthin improved cell viability in PC12 cells by diminishing the Aβ 1-42 peptides and the hydrogen peroxide-induced toxicity. Still, when compared to fucoxanthin, higher doses of astaxanthin were required to obtain a comparable effect, suggesting the former carotenoid might be more efficient.

On the other hand, astaxanthin’s molecular structure, with two hydroxylated groups, facilitates its interaction with PL polar heads and its integration in the PL bilayer, thus preventing lipid peroxidation but also promoting or maintaining the neural plasticity, that decreases during the ageing process [320].

Among meroterpenoids from marine origin, sargachromenol, sargahydroquinoic acid, and sargaquinoic acid are the most meaningful substances considered to possess anti-AD effects. They have been already extracted from several different species from brown seaweeds from the genus *Sargassum*. The scientific evidence produced so far enlightened some of the possible roles of these compounds in the prevention and/or treatment of AD. The anti-cholinesterase activity, an anti-inflammatory action, and the promotion of nerve growth factor, are some examples of the processes where sargachromenol, sargahydroquinoic acid, and sargaquinoic acid take part.

In this regard, Seong, et al. [139] described a potent inhibitory action of these compounds against the activities of BChE (with an IC_50_ of 9.4, 15.1 and 10.4 μM, in the same order) and BACE1 (IC_50_ of 6.9, 4.3 and 12.5 μM, respectively), along with moderate AChE inhibition. The kinetic study and molecular docking simulation led the authors to ascribe this inhibitory effect to the strong hydrogen bonds formed between sargachromenol, sargahydroquinoic acid, and sargaquinoic acid and the residues located in the catalytic aspartyl and allosteric sites of BACE1. A similar inhibitory pattern was seen by Choi, et al. [137] who studied the sargaquinoic acid and sargachromenol obtained from *S. sagamianum*. In this work, the IC_50_ value determined for AChE inhibition corresponded to 23.2 μM and 32.7 μM, while the BChE inhibition had an IC_50_ value of 26 nM and 7.3 μM, for sargaquinoic acid and sargachromenol, in the same order.

From another *Sargassum* species, the *S. micracanthum*, Yang and co-workers [141] have isolated sargachromenol, which intervened in induced inflammatory processes *via* the reduction in NO and PGE2 production, and likewise the downregulation of expression of iNOS and COX-2 protein. A similar effect regarding the NO production and iNOS expression was perceived in cells treated with sargaquinoic acid extracted from *S. siliquastrum* [253]. The anti-inflammatory effect observed for sargachromenol and sargaquinoic acid is likely to be related with the inhibitory-κB protein (IκB-α)/NF-κB and extracellular signal-regulated kinase (ERK)/JNK pathways [253,321].

Besides neuroprotection, compounds endowed with neuritogenic activity may deliver the potential to reconstruct the damaged neuronal network, induced in neurodegenerative diseases like AD [239]. Sargachromenol and sargaquinoic acid extracted from *S. macrocarpum* have both proved to have this ability, to promote neurite outgrowth, when combined with nerve growth factor [138,140].

Furthermore, it is worth mention that the anti-AD activity of these meroterpenoids may assume greater relevance, as there is evidence that the prenylation enhances the compounds lipophilicity, resulting in increased affinity with cell membranes [322], hence favouring the transport from enterocytes to the internal circulation and later transfer from the circulation to tissues [323].

### 4.3. Sterols

The brain contains approximately 25% of the body’s total cholesterol, accounting for 70% of its composition, particularly within neuronal cell membranes [324]. The connection between cholesterol and AD first emerged when studies observed a lower prevalence of AD in patients undergoing statin treatment [324]. Moreover, excessive neuronal cholesterol accumulation was linked to the formation of phosphorylated tau tangles [325], and disruptions in cholesterol homeostasis have been associated with heightened inflammation and AD progression [4,150,325].

Yet some authors highlight that more than altered levels alone, and an imbalance in cholesterol turnover rates and changes in intra- and intercellular cholesterol distribution in the brain may play a crucial role in neuropathologies like AD [152]. Within the brain, cholesterol metabolism is tightly regulated and the LXRβ is a key player in this process. Indeed, numerous studies have underscored the protective function of LXR in the molecular pathways linked to AD development. For instance, in mouse models overexpressing mutated APP, the genetic deletion of LXRα or LXRβ intensified brain deposits of Aβ40 and Aβ42 peptides [326]. Conversely, LXR activation was shown to significantly decrease soluble Aβ40 and Aβ42 peptide levels [327]. Liver X receptors contribute to cholesterol efflux, supplying neurons with essential lipids, facilitating synaptic plasticity, and supporting neuronal repair following injury [152]. In addition, LXR stimulation was found to reduce tau protein aggregation in tauopathy mouse models [328]. Consequently, targeting LXR and cholesterol metabolism is being explored as a potential strategy for AD prevention and treatment.

In this context, marine phytosterols have emerged as promising candidates for cholesterol regulation and homeostasis. These marine-derived sterols have been shown to lower cholesterol levels in the bloodstream [150], while also demonstrating anti-apoptotic, antioxidant, and anti-inflammatory properties as they have the ability to modulate cell survival pathways, including BDNF, Nrf2, and NF-κB signalling, thereby suggesting that they offer neuroprotection [4].

Among these sterols, fucosterol and saringosterol stand out as the most promising compounds for combatting AD-related neurodegeneration. Their actions target key disease biomarkers, including oxidative stress, neuroinflammation, cholinergic deficits, amyloid formation, cholesterol dysregulation, and neuronal survival pathways [4,329,330,331].

Research conducted by Castro-Silva, et al. [158], which evaluated fucosterol anti-AD potential using in vitro and in silico methodologies, revealed its capacity to inhibit AChE. Another study conducted by Gan, et al. [254] examined the impact of fucosterol by counteracting the Aβ peptides effect on SH-SY5Y cells, exposing them to 10 μM or 20 μM prior to Aβ peptides induction. The obtained results indicated that fucosterol counteracts Aβ peptides toxicity, reducing neurotoxic effects and Aβ peptides-induced apoptosis. Additionally, the pre-treatment with fucosterol downregulated APP mRNA expression and diminished intracellular Aβ peptides accumulation.

Further investigations suggest that fucosterol exerts neuroprotective effects via its AChE and BChE inhibitory activity, as well as its ability to mitigate LPS- and Aβ peptides-triggered neuroinflammation by suppressing pro-inflammatory cytokines, including IL-6, IL-1β, and TNF-α [157]. Studies conducted by Jung, et al. [237] revealed its anti-amyloidogenic properties, particularly in relation to BACE1, a pivotal enzyme in Aβ peptides production. This of particular importance as this enzyme represents the initial step in the amyloidogenic cascade. Moreover, findings indicate that fucosterol enhances hippocampal neuron viability in ageing rat models [156].

Several studies have emphasized the ability of saringosterol to activate LXR [154,199,255], making it a promising candidate for cholesterol homeostasis regulation in therapeutic applications. Interestingly, findings from Chen, et al. [154] and Zhan, et al. [255] suggest that the bioactive potential of saringosterol may vary depending on its isomeric form, with 24(S)-saringosterol exhibiting markedly higher LXR activation rates in HEK-293T and SH-SY5Y cells, respectively, when compared to 24(R)-saringosterol.

The neuroprotective effects of saringosterol have been further demonstrated in in vivo studies using APPswePS1∆E9 mice, a commonly used AD mouse model. In research conducted by Martens, et al. [200], 6-month-old mice received a daily oral dose of 0.5 mg 24(S)-saringosterol per 25 g of body weight for a period of 10 weeks. Furthermore, treatment helped restore microglial activation and inflammation markers, including the expression of ionized calcium binding protein 1, bringing microglia numbers in AD mice back to levels similar to wild-type mice [200]. Despite these improvements, Aβ peptides plaque burden remained unchanged, though cognitive decline prevention was observed.

Similarly, Bogie, et al. [199] reported cognitive improvements in AD mice following dietary supplementation with either dried *S. fusiforme* or a lipid extract derived from the same seaweed, with a notable enhancement in short-term memory. However, in contrast to Martens, et al. [200], Bogie, et al. [199] found that supplementation led to a substantial 81% reduction in hippocampal Aβ peptides plaque load. This discrepancy may stem from the presence of additional bioactive compounds within the seaweed or its lipid extract, beyond just saringosterol, that may have contributed to Aβ peptides clearance. Consequently, an approach based on whole seaweed or its extracts might be more effective for AD management, potentially benefiting from synergistic interactions with other phytosterols (such as sitosterol and stigmasterol) and carotenoids (like fucoxanthin), which are naturally abundant in brown seaweed [200].

## 5. Health Impacts

Given the major health issue represented by AD and the difficulty in treating the disease once detected, preventive approaches have been proposed. Among these, the application of specific nutraceutical ingredients has been receiving significant attention [332]. Although evidence on health impacts remains inconclusive, the diversity, bioavailability, and established health benefits of marine lipids (see Section 2) suggest possible effects on neuronal tissues.

In this sense, different studies may be carried out, ranging from clinical, epidemiological, purely observational to interventional, and from in vitro assays to in vivo models, making it hard to compare directly and draw firm conclusions. There are also different levels of strength of the existing evidence. This may be classified as convincing, probable, possible, and insufficient [333]. Of course, when a wide range of different studies of various types unanimously point to a given causality or correlation, this observation may be considered convincing evidence. However, in the domain of the health impacts of dietary options or components, this only happens in very few cases. There are too many confounding factors and the studies very often lack enough representativeness, robust experimental design, and sound statistical treatment [334,335]. Moreover, many studies do not concern health endpoints, but intermediate points considered diagnostic for AD. These may be Aβ peptides-induced neurotoxicity and associated oxidative stress, apoptosis, neuroinflammation, and levels of hyper-phosphorylated tau protein [225]. All these different kinds of studies have to be considered for an overall evaluation of the impact of marine lipids in AD.

### 5.1. Phospholipids

The biochemical diversity of PL represents an additional hurdle in the investigation into the associations between specific PL molecules and AD. On the one hand, the additional group attached to the phosphate and, on the other, the specific esterified FA add significant layers of complexity onto the research in this field. In spite of some inconclusive studies, some general trends seem to be identifiable and may be linked to well-known mechanisms.

In their in vitro experiments Hans, et al. [214], for example, found a beneficial effect of PL extracted from salmon fillet over some of these mechanisms, particularly by lowering the generation of ROS or the extracellular Aβ peptides accumulation. Another effect noted by these authors is that salmon PL acted over astrocyte regulation and reduced their activation. Astrocytes are essential glial cells involved in a wide range of physiological processes within the brain, such as supplying energy to neurons, and the BBB maintenance [336]. Yet, astrocytes are also believed to play an ambivalent role over Aβ peptides: while they can breakdown Aβ peptides [337], in response to inflammation they suffer functional and morphological changes becoming reactive and release pro-inflammatory cytokines and large quantities of these peptides, thus contributing to the acceleration of AD progression [214,336].

As discussed previously in Section 4.1, PLs, such as PC, may be effective in reducing neuronal inflammatory activities through the inhibition of MAPK and other signalling systems [219]. Such activities and triggering of specific systems affect neurodegeneration in AD, thus suggesting supplementation with dietary PLs as a means of modulating neuronal composition and as a potential therapeutic strategy for AD. It is worth mentioning that a choline-supplemented diet started early on has been claimed to lead to long-term improvements in cognitive function [338,339]. However, there are clinical studies that did not find beneficial effects of PL (especially PC) on AD, thereby justifying more investigation for achieving a deeper understanding of AD etiology and possible dietary strategies to prevent and delay the onset of AD [29].

Phosphatidylserine is one of the most studied PL regarding brain health, thereby encompassing thousands of scientific papers and, in particular, a large number of randomized, double blind, placebo-controlled clinical trials [340]. It has been alleged that PS can help in improving memory, learning, concentration, and even vocabulary skills, its effect on the stimulation of ACh synthesis—triggering the release of neurotransmitters from the vesicles into the synaptic gap—being a possible route for such improvements [29,220]. This is supported by several double-blind, placebo-controlled clinical trials assessing the outcome of PS treatment in patients affected by age-related cognitive decline and/or early AD as revised by Louis-Sylvestre [341]. The data from these studies indicated that PS supplementation in the diet led to substantial enhancement in cognition and memory functions with respect to the placebo [29,341]. An open-label extension study demonstrated that consumption of 100 mg/day of PS-DHA could improve or maintain cognitive status in elderly individuals with memory complaints [220]. These studies are also supported by other biochemical findings [64] pointing to the protection of PC12 cells—a cell line with an embryonic origin from the neural crest composed by a mixture of neuroblastic cells and eosinophilic cells—from oxidative stress and prevention of mitochondrial-mediated apoptosis by EPA/DHA-PS. Moreover, Che, et al. [64] were able to show through the assessment of cell viability and the results for the leakage of lactate dehydrogenase that the neuroprotective effect of PS was higher than that of PC.

The combination of PC and PS is also another possibility. In this regard, Zhou, et al. [221] showed that the administration of DHA-PC and DHA-PS in SAMP8 mice models of AD ameliorated cognitive deficits by affecting in a positive way Aβ peptides pathology, mitochondrial damage, neuroinflammation, neurotrophic factors, and oxidative stress.

The effect of PLG on AD patients has also been the subject of various studies, including clinical trials. Ethanolamine PLG, a very relevant PL in neuronal membranes—60 to 90% of total PE in molar terms—is specifically reduced in brains from patients with AD [342]. Yamashita, et al. [48] investigated how EtnPLG administration could affect cognitive deficits and lipid composition in rats, an animal model of AD. In the trial conducted, there were three groups: Control, Egg, and Ascidian [48]. The Control group only received orally administered vehicle, the Egg group was given 260 μmol PE/kg body weight (bw)/day and 10 μmol as EtnPLG/kg bw/day, and the Ascidian group received also 260 μmol PE/kg bw/day, but 209 μmol as EtnPLG/kg bw/day [48]. The last group, rats administered ascidian preparation (rich in EtnPLG), showed benefits in both reference and working memory-related learning abilities. Though total PLG concentration did not show differences, the Ascidian group had significantly higher levels of 18:0ol/22:6-EtnPLG in the cerebral cortex [48]. These levels of 18:0ol/22:6-EtnPLG were correlated with the working memory-related learning ability. In humans, a multi-centre, randomized, double-blind, placebo-controlled trial of 24 weeks with 328 patients aged 60 to 85 years as participants [222] was carried out in Japan. The participants in this study received either 1 mg/day of PLG (purified from scallop) or placebo. In mild AD patients, Wechsler Memory Scale-Revised scores improved significantly in the treatment group [222]. More specifically, Wechsler Memory Scale-Revised scores significantly improved among females and those older than 77 years in the treatment group. It should be remarked that scallop-derived PLG are known to be relatively rich in EPA and DHA [343].

All the aforementioned studies used natural PL, which are almost always mixtures of different molecules, at least at the FA level. Hence, synthetic PL with previously conceived specific structures may be advantageous. In this context, structured PL, such as AceDoPC, have been tested with some promising results. In fact, Lagarde, et al. [58] reported that AceDoPC prevents more efficiently the negative effects of experimental stroke on rats than the unesterified DHA. In addition, these authors observed that AceDoPC inhibits PAF-induced human blood platelet aggregation. From such observations, it was deduced that AceDoPC could potentially operate as an efficient DHA transporter to the brain and as a neuroprotective agent [58]. On the other hand, Fourrier, et al. [223] claimed that a single injection of AceDoPC lessened the LPS-induced neuroinflammation by promoting a reduction in IL-6 production. Moreover, neural stem progenitor cells subjected to hypoxemic conditions in vitro and treated with AceDoPC presented enhanced neurogenesis if compared to unesterified DHA under pathological conditions [224]. All these studies highlight the importance of binding DHA to appropriate specific positions in larger biochemical structures.

More than the group attached to phosphate in the PL structure, the particular FA in the PL molecule may be decisive for the health outcome [344]. Namely, Wen, et al. [225] reported that EPA-PL improved Aβ 1-40 peptides-induced cognitive deficiency in rats. These authors also found that EPA-PL alleviated Aβ peptides-induced neurotoxicity including oxidative stress, apoptosis, neuro-inflammation cascade, and hyper-phosphorylated tau in a dose-dependent pattern. According to a recent study encompassing cell viability and leakage of lactate dehydrogenase [64], DHA/EPA-PC and -PS were found to be superior to PC and PS without DHA or EPA. Furthermore, the improvement in these parameters with n-3 PUFA-enriched PS was also dose-dependent [64]. Furthermore, Zhang, et al. [40] applied marine PL (a mixture of PC, PI, and PE, among others) rich in palmitic acid and DHA to the supernatant of Chinese hamster ovary cells (transfected with APP and PS1), in order to determine the level of secretion of amyloid beta Aβ 1–42. The results of this in vitro study showed that, in comparison to the model control group, Aβ 1–42 peptides were reduced by 69% with the tested marine PL [40]. Other studies are further afield and are carried out in human subjects. A 24-week, randomized, double-blind placebo-controlled study [345] enabled testing the possibility of using n-3 PUFA monotherapy (1080 mg of EPA and 720 mg of DHA obtained from menhaden fish body oil concentrate) in individuals with cognitive impairment and to analyze its effects on cognitive function. There was no difference in the cognitive portion of the AD Assessment Scale change during follow-up in the n-3 PUFA treatment and placebo groups [345]. However, for those individuals with MCI, n-3 PUFA group showed improvement in cognitive impairment in comparison to the placebo group. An overall meta-analysis considering total FA, SFA, MUFA, PUFA, n-6 PUFA, and n-3 PUFA [346] only showed a significant association between this last parameter and MCI risk, thus reinforcing the importance of the action of n-3 PUFA-containing PL against AD.

Among marine natural molecules, there are some organisms with very high levels of PL rich in n-3 PUFA. Krill oil rich in DHA-PL has been considered to favour a better balance of low- and high-density lipoproteins [49]. In fact, a meta-analysis of data from seven eligible trials with 662 participants showed that krill oil supplementation is effective in reducing plasma concentrations of LDL and TAG, while ensuring a higher concentration of HDL [347]. The effect of this dietary option may be deeper and more extensive as suggested by a study on the potential of krill oil in improving memory function in rats [226]. Krill PL fostered the development of both short- and long-term memories [226]. Concomitantly, in the cerebral cortex and hippocampus and with respect to the control group, while the DHA level was significantly increased, the arachidonic acid level was reduced [226]. In addition, administration of krill PL led to a reduction in lipid peroxides in the plasma and brain as well as of ROS in the cerebral cortex and hippocampus. Finally, Gamoh, et al. [226] observed that special memory was improved with high krill PL intake.

There is still much debate concerning the role of particular long-chain n-3 PUFA and, more specifically, the particular importance of EPA and DHA in the prevention and mitigation of AD. Hence, there has been much research on the effects of EPA and DHA as FFA, either alone or in combinations of 1:1, 1:2, and 2:1 on cell models of AD [348]. The experimental outcomes indicate that both long-chain n-3 PUFA attenuate neuron apoptosis and enhance cell viability with synergistic anti-inflammatory action in the AD model [348]. However, whereas non-esterified EPA showed to be more effective against oxidative stress, DHA alone demonstrated a higher capacity to improve neurotrophic systems [348]. Regarding this issue, it is worth noting that DHA content in the central nervous system decreases with age and this phenomenon has been correlated with senile dementia or AD [349].

In any case, for an effective action of n-3 PUFA upon health endpoints, the chemical structure in which they are bound does matter, with PL being considered more effective than other forms, such as TAG or ethyl ester [49]. This effectivity may be related to the transport of the n-3 PUFA to the brain. Though tracer studies indicate that DHA-PL is more effective at targeting the brain than DHA-TAG, a differentiated DHA accretion was not detected by Kitson, et al. [350]. These authors claimed that DHA-TAG, DHA-PL or a mixture of both were all equally effective at augmenting brain DHA content. Kitson, et al. [350] summarized their findings in the dichotomy of more labelled DHA entering the brain when consumed as PL—this may also apply to synthetic PL [61]—but failing to translate into higher brain DHA levels. This is controversial. For instance, in obese Zucker rats, Di Marzo, et al. [351] observed that supplementation with n-3 PUFA in PL structure from krill oil augmented the levels of DHA in the brain more than the alternative supplementation of n-3 PUFA in TAG. Indeed, a substantially higher incorporation of EPA and DHA into brain PL after four weeks of krill oil supplementation in contrast to fish oil (predominantly TAG) was recorded [351].

From the majority of the experimental works and clinical trials, it seems to be clear that dietary PL rich in n-3 PUFA, have a variable positive effect on AD, apparently without severe side effects. PS molecules enriched in DHA and, possibly, other n-3 PUFA may deserve to be a research focus for stronger benefits in the prevention of AD. In any case, studies show that PL molecules per se are not sufficiently effective against AD to be considered as a single future therapy, but they may be part of preventive approach combining diet and medicines, especially in individuals with particular genetic traits that predispose to disease or weaken the resistance of the neuronal system to the onset of AD.

### 5.2. Terpenoids

Many terpenoids have been shown to exhibit a broad spectrum of biological activities with pharmacological applications, including antimicrobial, anticancer, antiviral, anti-inflammatory effects [68]. More specifically, in what concerns to AD, several terpenoids obtained from marine organisms have been reported to display anti-inflammatory, anticholinesterase, anti-amyloidogenic and aggregation inhibition activities, as well as neuroprotective effects and improvement in cognitive function [103,239,252,291,292,293].

A wide variety of sesquiterpenoids have been already investigated as compounds endowed with biological activities that might ameliorate and delay the AD symptoms onset. Found in marine seaweeds or other marine organisms like molluscs, these group of compounds include, for example, α-bisabolol, aplysistatin, and caulerpenyne. Fernandes, et al. [228] in their investigation evaluated the impact of α-bisabolol treatment in mice with induced ischemia. The authors related the treatment with 100 and 200 mg/(kg·day) of this sesquiterpenoid with the prevention of memory deficits, improvements in short- and long-term memory, significant prevention in the loss of recognition abilities, as well as the prevention of spatial memory impairments.

It is known that vitamin A and its derivatives have an important role in what concerns neuronal health, namely on adult neuronal functioning, memory, and neuronal plasticity [352]. In this regard, retinol was linked with learning capacity, memory and sleep, schizophrenia, depression, Parkinson disease, and AD by Tafti and Ghyselinck [353]. For instance, the deficiency in vitamin A was correlated with augmented cognitive decline in the elderly population [305]. More recently, in the population-based study involving 1817 participants aged 50 and above, carried out by Li, et al. [307] it was concluded that plasma retinol levels of ≥0.539 μg/mL, well above the World Health Organization’s current recommendations of ≥0.300 μg/mL in adults, were negatively correlated with the risk of mild cognitive impairment. Likewise, Craft, et al. [354] have observed an age-related decline in retinol levels in the frontal lobes. Additionally, the levels of vitamin A and the provitamin A (β-carotene) in serum and plasma of AD individuals are significantly reduced [103]. On the other hand, the treatment with retinoic acid rescued the memory deficits of AD model mice that denoted improved spatial learning and memory compared to those who were not treated [230].

There is strong scientific evidence relating the deficiency of vitamin D with neurological disorders and compromised cognitive function [355]. In the literature, there can be found several observational and longitudinal investigations dealing with low vitamin D serum levels that have positively related to this condition with the enhanced risk of dementia and cognitive decline in general and in AD [356,357,358].

With the objective to enlighten the impact of vitamin D on cognitive function during ageing, Latimer, et al. [235] carried out an interventional study with F344 rats. The authors started the experiment at middle age (when, similarly to humans, the first markers of ageing can be perceived) and simulated three levels of vitamin status: deficient, sufficient, and higher. According to their results, only rats on higher doses presented vitamin D serum levels in the optimal range and could perform a complex memory task, when compared to low- or normal-dietary vitamin D groups. Hence, these results confirmed the relation between calcitriol serum levels and its protective action over the cognitive function. The authors also pointed out the importance of the timing of the intervention, considering that the supplementation at earlier stages should be more effective in promoting healthy cognitive ageing. Another investigation conducted by Briones and Darwish [232] using aged and young F344 rats led concluded that vitamin D supplementation ameliorated the age-related cognitive impairment, leading to improvements in learning ability and memory in aged animals. In contrast, no beneficial effects were observed in young rats.

So far, some clinical trials have also been undertaken in order to look up for the potential benefit of vitamin D supplementation in patients diagnosed with AD. In their investigation Annweiler, et al. [359] attained results that allowed them to establish a significant correlation between the patients’ dietary intake of this vitamin and the onset of AD. Moreover, the lowest vitamin dietary intakes (up to 77.72 μg/week) were associated with increased risk to develop AD. On the contrary, patients with the highest vitamin D dietary intakes (77.72–205.54 μg/week) showed a lower risk of AD.

Benefits were also perceived when the supplementation with vitamin D was combined with memantine, a drug commonly used for the treatment of AD, where patients revealed a statistically and clinically relevant gain in cognition [236]. For this study, the 43 participants were distributed by three groups that were treated with: (1) memantine, (2) vitamin D, (3) memantine and vitamin D. For a 6-month period, individuals received doses of 20 mg/day of memantine, while vitamin D supplements were orally taken on a daily or monthly basis. Doses between 400 and 1000 IU/day, or 100,000 and 200,000 IU/month of vitamin D, were prescribed to the participants. Considering that the combination proved to be more effective than both substances alone, Annweiler, et al. [236] hypothesized about a possible synergistic effect when vitamin D and memantine are taken together.

Carotenoids comprise another group of compounds often related with neuronal tissue health and a better cognition status in elderly. The investigation carried out by Johnson, et al. [360], is a good example of this, the authors being able to demonstrate the link between the serum and brain levels of carotenoids and the cognitive function in the whole population, but also among octogenarians and centenarians. Indeed, this study highlighted the significant correlation between the dietary carotenoids, estimated both in the serum and in the brain with measurements of cognitive function. These findings were also in agreement with those reported by Perrig, et al. [361].

In particular, there is already consistent scientific evidence showing that the treatment with fucoxanthin has positive effects on mice with induced cognitive impairment. Through Morris Water Maze test, both Xiang, et al. [116] and Lin, et al. [238] have observed significant improvements in terms of mice recognition performance, as well as spatial learning and memory impairments. According to Dhami, et al.’s [240] findings, a 14-day treatment with fucoxanthin improved cognitive functions in Wistar rats induced with significant impairment in learning and memory through the intracerebroventricular infusion of streptozotocin. Jiang, et al. [241], on the other hand, evaluated the benefits of a long-term treatment through an in vivo trial with APP/PS1 transgenic mice that were treated with fucoxanthin twice a week for a total of 20 weeks. In their study, the administration of this carotenoid prevented the cognitive deficits and the Aβ peptides-related neuroinflammation well known in this animal model.

Even though fucoxanthin is able to cross the BBB and accumulate in the brain, its application as pharmaceutical or nutraceutical, however, faces an important drawback due to its low bioavailability, which is believed to be due to its conversion to fucoxanthinol in the plasma [362]. Considering this, some strategies have been tested in order to improve fucoxanthin bioavailability: namely dietary combination of fucoxanthin and edible oil or utilization of fortified skimmed milk [317,363]. Yang, et al. [364] loaded nanoparticles of poly lactic-*co*-glycolic acid-*block*-polyethylene glycol with fucoxanthin and observed that they were more efficient in preventing cognitive impairment usually observed in Aβ peptides soligomers-induced AD mice, when compared to fucoxanthin free form.

Rahman, et al. [243] investigated the potential of oral administration of astaxanthin in Wistar rats previously induced with AD. By using Morris Water Maze and Novel Object Recognition tests, the authors observed that astaxanthin reverted these animals cognitive and memory impairment. More importantly, Rahman and colleagues found the benefits were dose-dependent. Han, et al. [246] also reported that astaxanthin ameliorated LPS-induced memory loss. Similarly, another study with aged mice fed with an astaxanthin-enriched diet, for one month, showed an improved performance on hippocampal-dependent cognitive tasks [247]. Astaxanthin also contributed to ameliorate behavioural disorders, spatial learning and memory in a ferrous amyloid buthionine-infused sporadic AD rat model [248].

Clinical trials including astaxanthin also seem to support the benefits of this xanthophyll in the reversion of neuronal functions related with the ageing process [365]. A clinical study involving 96 individuals with self-reported complaints of age-related forgetfulness, revealed that supplementation with astaxanthin-rich *H. pluvialis* extracts, with 6 and 12 mg/day, improved the participants’ memory scores [249].

The randomized, double-blind, placebo-controlled trial undertaken by Ito, et al. [251] supplemented a group of 21 individuals with 50 to 79 years old, with mild cognitive impairment, using a combination of astaxanthin and sesamin (also endowed with neuroprotective activity). For this study, the authors hypothesized that the combination of these substances could exert a stronger anti-oxidative activity than single supplementation. Participants took 2 capsules/day of the supplement containing 3 mg of astaxanthin and 5 mg of sesamin, for 12 weeks. The cognitive tests displayed improvements in psychomotor speed and processing speed, leading the authors to conclude that the supplementation ameliorated the participants’ cognitive functions.

The clinical trial carried out by Hayashi and colleagues [250], in turn, included a group of 54 participants, with ages ranging between 45 and 64, that were supplemented with 8 mg/day of astaxanthin for 8 weeks. After this period, the cognitive tests showed that participants on supplementation with astaxanthin had significant improvements from their baseline, when compared to placebo group. Hence, all these studies show promising therapeutic possibilities associated with different groups of terpenoids present in marine resources, with more synergistic studies encompassing compounds of different classes, of marine origin or not, advisable for achieving the maximal effect on AD prevention and mitigation.

### 5.3. Sterols

Several trials focused on the neurodegeneration management through dietary strategies rich in fucosterol and saringosterol have underlined the pharmacological potential of these phytosterols against neurodegenerative diseases.

A study carried out in ageing rats that have been unilaterally infused with fucosterol, into the dorsal hippocampus at a rate of 10 µmol/h for 4 weeks showed this sterol counteracted the Aβ1-42 peptides-induced neurodegeneration, ameliorating both the viability of hippocampal neurons and cognitive impairment [156].

The dietary supplementation of animal models for AD (APPswePS1ΔE9) with *S. fusiforme* proved to be beneficial in delaying the cognitive decline in this AD model [199]. In this trial, *S. fusiforme* was administrated in two different ways: pulverized dried in the chow (estimated intake of 242 µg/day for 10 weeks) or as a lipid extract by oral gavage (664 µg 24(S)/(R)-Saringosterol/day for 6 weeks). Following this treatment, the behavioural tests undertaken by Bogie, et al. [199] showed that AD mice significantly improved their short-term memory, irrespective of if *S. fusiforme* was taken in the provided chow or as lipid extract.

The prevention of the cognitive decline, without adverse effects on liver fat content, was also accomplished in APPswePS1∆E9 mice that received a daily oral gavage dose of 0.5 mg 24(S)-saringosterol per 25 g body weight for 10 consecutive weeks [200]. Interestingly the benefits in terms of cognition did not correlate with the decrease Aβ peptides load [200]. A similar trend was reported in a more recent investigation, where APPswePS1∆E9 mice diets were supplemented with a lipid extract of *H. elongata* (192 µg/g saringosterol and 3760 µg/g fucosterol) or supercritical fluid extract of *S. fusiforme* (304 µg/g saringosterol and 4797 µg/g fucosterol), and the collected data demonstrate the prevention of cognitive decline for both groups without a reduction in cerebral amyloid-β plaque load. Based on these findings, it was proposed that insoluble Aβ peptides plaques may not be the direct cause of clinical symptoms, suggesting that reducing Aβ peptides plaque accumulation may not be essential for improving AD symptoms [153].

It would be interesting to corroborate such results in humans, but as far as the authors are aware, there are no human clinical trials specifically investigating fucosterol or saringosterol for AD.

## 6. Conclusions

The extensive bibliographic review conducted for this work highlights the significance of ongoing efforts to identify a wide variety of marine bioactive compounds with potential anti-AD properties, as well as new organisms capable of producing these biomolecules. In particular, this review has compiled numerous phospholipids, terpenoids, and sterols that contribute to the reduction in several biomarkers associated with AD.

Even though marine-derived products may also contain lipophilic contaminants and pollutants, which should be carefully considered in the context of their safety and potential therapeutic application, the compounds addressed here demonstrated beneficial effects, such as enhancing the incorporation of crucial FA for neuronal health, reducing oxidative and inflammatory status, and mitigating cellular apoptosis. Moreover, their neuroprotective potential appears to influence the expression of BDNF, anti-cholinesterase activity (AChE and BChE), as well as cholesterol homeostasis.

This review highlights the pivotal role of marine lipids in neuroprotection through the multiple mechanisms relevant to AD. Although algae-derived oils often show higher bioaccessibility than fish oils, both sources provide physiologically meaningful amounts of long-chain n-3 PUFA. Their overall effectiveness, however, depends on factors such as molecular form, digestion, absorption, and tissue bioavailability, which supports the need for further research integrating biochemical, metabolic, and clinical evidence.

Although existing data on the bioaccessibility and/or bioavailability of these lipids suggest promising therapeutic potential, clinical trials demonstrating their neuroprotective effects in humans remain scarce or non-existent.

Whether used individually or in combination to achieve a broader therapeutic effect, marine lipids—both formulated as dietary supplements or incorporated as ingredient in drugs as anti-AD therapeutics—could play a fundamental role in maintaining neuronal health. In doing so, they may help prevent and decrease the progression of neurodegenerative diseases, including AD.

## Figures and Tables

**Table 1:** Classification of terpenoids from marine origin.

Classes	Isoprene Units	Examples
Monoterpenoids (C_10_H_16_)	2	pinene, limonene
Sesquiterpenoids (C_15_H_24_)	3	caulerpenyne; zonarol; (5E,10Z)-6,10,14-trimethylpentadeca-5,10-dien-2, 12-dione; (5E,9E,13E)-6,10,14-trimethylpentadeca-5,9,13-trien-2,12-dione
Diterpenoids (C_20_H_32_)	4	vitamin A (retinol)
Sesterpenoids (C_25_H_40_)	5	manoalide
Triterpenoids (C_30_H_48_)	6	glycyrrhizin, 18α-glycyrrhetinic acid, 18β-glycyrrhetinic acid, vitamin D (cholecalciferol), squalene
Tetraterpenoids (C_40_H_64_)	8	fucoxanthin, fucoxanthinol, astaxanthin, lycopene, lutein, zeaxanthin, β-carotene
Meroterpenoids	2	sargachromenol, sargaquinoic acid, sargahydroquinoic acid

**Table 2:** Summary of the marine-derived compounds, including their proposed mechanisms of action and reported effects associated with AD pathology.

Lipid Class	Compound	Mechanism of Action/Health Impact	Reference
Phospholipids	unespecified	Decreased (↓) reactive oxygen species (ROS), ↓ Aβ peptides accumulation, astrocyte regulation	[214]
	EPA-PC DHA-PC	↓ Aβ peptides levels in amyloid precursor protein (APP) and presenilin 1 (PS1) (CHO-APP/PS1 cells) and SAMP8 mice, ↓ APP, PS1, and beta-site amyloid precursor protein cleaving enzyme (BACE1) expression, ↓ Apoptosis (via increased [↑] B-cell lymphoma 2 regulator proteins and ↓ expression of pro-apoptosis factors), ↑ brain-derived neurotrophic factor (BDNF), synaptophysin and growth associated protein 43 expression	[44]
	PLG	↓ microglial expression of protein kinase C delta (PKCδ), ↓ p38 mitogen-activated protein kinase (MAPK) and c-JUN N-terminal kinase (JNK) protein expression	[51]
	Phosphoinositide (in connection with scyllo-inositol)	↓ Aβ peptides aggregation	[215]
	Cardiolipin (CL)	homeostasis of the central nervous system	[216]
	EPA-Enriched Phosphoethanolamine PLG	↑ tropomyosin receptor kinase B/extracellular signal-regulated kinases/cyclic adenosine monophopsphate (AMP)-dependent response element-binding protein signalling pathway, ↓ oxidative stress and apoptosis	[217]
	DHA-PL (DHA-PC, DHA-PS)	↓ apoptosis via mitochondria-mediated pathway and MAPK pathway, ↑ dopaminergic neurons	[218]
	Dilinoleoylphosphatidylcholine	↓ induction of MAPK, prevents phosphorylation and activation of nuclear factor-kappa B, ↓ APP in SH-SY5Y cells	[219]
	PS-DHA	Improvement or maintenance of cognitive status in elderly subjects with memory complaints	[220]
	EPA/DHA-PS	Protection for oxidative stress and prevention of mitochondrial-mediated apoptosis in PC12 cells	[64]
	DHA-PC and DHA-PS	Improvement of oxidative stress, ↓ Aβ peptides levels, ↓ APP and BACE1 expression, ↓ interleukin 1 (IL-1) and tumour necrosis factor-alpha (TNF-α), ↓ Apoptosis, improvement on the decline of learning and memory in SAMP8 mice	[221]
	EtnPLG	Improvement in reference and working memory-related learning abilities	[48]
	PLG	Improvement in Wechsler Memory Scale-Revised scores in mild AD patients	[222]
	acetyl-LysoPC-DHA (AceDoPC)	↓ LPS-induced IL-6 production in C57Bl6/J mice hippocampus	[223]
		↑ neurogenesis in neural stem progenitor cells derived from the adult mouse brain	[224]
	EPA-PL	Improvement of Aβ peptides-induced cognitive deficiency, ↓ oxidative stress, ↓ apoptosis, ↓ neuro-inflammation cascade and tau hyper-phosphorylation in rats	[225]
	PS and PC	↑ cell viability, furthermore DHA/EPA-PS ↑ superoxide dismutase, ↑ total antioxidant capacity in PC12 cells	[64]
	PL mixture containing PC, PI, PE, PS Lyso-PC and Lyso-PE	↓ Aβ peptides secretion in SH-SY5Y cells	[40]
	unspecified	↓ Lipid peroxidation in the plasma and in brain, ↓ ROS in cortex and hippocampus, improvement on spatial-memory in rats	[226]
Terpenoids	Halogenated monoterpenoids	↓ acetylcholinesterase (AChE) activity	[77]
	α-bisabolol	↓ AChE and butylcholinesterase (BChE) activity, ↓ aggregation of Aβ peptides oligomers, ↓ disaggregation of mature fibrils, rescuing effect against Aβ peptides induced toxicity	[84,227]
	α-bisabolol	Beneficial in terms of the memory deficits, short and long-term memory, loss of recognition abilities and spatial memory impairments in mice	[228]
	Aplysistatin	↓ inflammation, ↓ nitric oxide (NO) and prostaglandin-E2 (PGE2), ↓ inducible nitric oxide synthase (iNOS) and cyclooxygenase 2 (COX-2) expression	[80]
	Caulerpenyne	↓ lipoxygenases (LOX) activity and unsaturated FA oxidation	[229]
	Zonarol	↓ cell death, activation of Nuclear factor (erythroid-derived 2)-like 2/antioxidant-responsive element (Nrf2/ARE) pathway	[86]
	Dihydromonofarnesylacetone, monooxofarnesylacetone	↓ AChE and BChE activities	[87]
	Retinol	↓ Aβ peptides deposition and tau phosphorylation, ↓ Aβ peptides induced toxicity	[230]
	Retinol	↓ tau aggregates, ↓ cyclin-dependent kinase 5 and glycogen synthase kinase 3β, ↑ astrocytes and microglia	[231]
	Retinoic acid	rescued of memory deficits, improved spatial learning and memory in mice	[230]
	Vitamin D	Improvement in age-related decline in learning and memory, age-related change in inflammatory status was counteracted, ↑ Aβ peptides clearance, ↓ amyloid burden in aged rats	[232]
		↓ Aβ peptides-induced reactive oxygen species, apoptosis, and tau protein hyperphosphorylation	[233]
		↓ Aβ peptides levels, improved conditioned fear memory	[234]
		protection of cognitive function in rats	[235]
	vitamin D (combined with memantine)	enhanced cognition in AD patients	[236]
	Fucoxanthin	↓ BACE1 activity and Aβ peptides accumulation	[237]
		↓ BChE activity	[118]
		↓ AChE activity and BDNF expression, improvement on cognitive impairment in mice	[238]
		↓ Aβ peptides fibrils and oligomers, ↓ neurotoxicity of Aβ peptides oligomers in SH-SY5Y cells, and ↓ oxidative stress, ↑ expression of BDNF, improvement on cognitive impairment in mice	[116]
		↓ secretion of proinflammatory mediators: iNOS, COX-2, phosphorylation of MAPK, TNF-α, IL-6, IL-1β, and PGE2	[239]
		Improvement on cognitive functions in rats	[240]
		prevention of cognitive decline and Aβ peptides-related neuroinflammation as well as the secretion of pro-inflammatory cytokines and the activation of BV2 microglial cells	[241]
	Fucoxanthin and astaxanthin	↓ Aβ peptides aggregation, ↓ Aβ peptides induced cytotoxicity and apoptosis	[242]
	Astaxanthin	↓ Aβ peptides aggregation, ↓ Aβ peptides induced cytotoxicity and apoptosis	[242]
		anti-amyloidogenic effect, ↓ TNF-α level, ↓ AChE activity, ↓ oxidative stress, reversion of cognitive and memory impairment	[243]
		↓ ROS, ↓ SH-SY5Y and PC12 cells apoptosis, ↓ iNOS and COX-2, ↓ IL-6 and the MAPK	[132,244,245]
		reversion of the cognitive and memory impairment, ↓ levels of Aβ peptides, TNF-α, AChE, nitrite and oxidative stress	[243]
		↓ lipopolysaccharide(LPS)-induced oxidant activity, neuroinflammatory response and amyloidogenesis in microglia BV-2 Cells, and alleviates memory impairment in mice	[246]
		enhanced performance on hippocampal-dependent cognitive tasks in mice	[247]
		↓ neuroinflammation and ameliorate behavioural deficits in rats	[248]
		improved memory scores in individuals with self-reported complaints of age-related forgetfulness	[249]
		Improvement on cognitive function in middle-aged and older individuals	[250]
	Astaxanthin (combined with sesamin)	Improvement of cognitive functions in individuals with mild cognitive impairment (MCI)	[251]
	Fucoxanthinol	Antioxidant activity	[102,239,252]
	Sargachromenol, sargahydroquinoic acid, and sargaquinoic acid	↓ AChE, BChE and BACE1 activities	[139]
	Sargaquinoic acid and sargachromenol	↓ AChE and BChE activities	[137]
	Sargachromenol	↓ NO and PGE2, ↓ iNOS and COX-2	[141]
	Sargaquinoic acid	↓ NO and iNOS	[253]
Sterols	Fucosterol	↓ BACE1 activity and Aβ peptides accumulation	[237]
		↓ AChE activity	[158]
		↓ Aβ peptides accumulation and ↓ Aβ peptides-induced apoptosis in SH-SY5Y cells	[254]
		↓ AChE and BChE activities, ↓ LPS- and Aβ peptides-triggered neuroinflammation via suppression of IL-6, IL-1β, and TNF-α	[157]
		↑ Hippocampal neuron viability and cognitive impairment in ageing rat models	[156]
	24(S)-Saringosterol	Activation of liver X receptor beta (LXRβ) in microglial cells, ↑ short-term memory, ↓ hippocampal Aβ peptides plaque load in APPswePS1ΔE9 mice	[199]
		Activation of LXRβ	[154]
		Activation of LXRα and LXRβ in SH-SY5Y cells	[255]
		Prevention of cognitive decline and the increase in the inflammatory marker Iba1 in mice cortex	[200]

↓—decreased, ↑—increased.

## Data Availability

Dataset available on request from the authors.

## Abbreviations

The following abbreviations are used in this manuscript:

AceDoPC—acetyl-LysoPC-DHA, ACh—acetylcholine, AChE—acetylcholinesterase, AD—Alzheimer’s disease, AMP—adenosine monophopsphate, APOE ε4—apolipoprotein E type 4 allele, APP—amyloid precursor protein, ARE—antioxidant-responsive element, ATP—adenosine triphopsphate, Aβ—beta-amyloid, BACE1—beta-site amyloid precursor protein cleaving enzyme, BBB—blood–brain barrier, BDNF—brain-derived neurotrophic factor, BChE—butyrylcholinesterase, CER—cerebroside, CL—cardiolipin, CM—ceramide, COX—cyclooxygenase, DAG—diacylglycerol, DHA (22:6 n-3)—docosahexaenoic acid, DMAPP—dimethylallyl diphosphate, DW—dry weigh, EPA (20:5 n-3)—eicosapentaenoic acid, ERK—extracellular signal-regulated kinase, EtnPLG—Ethanolamine PLG, FA—fatty acids, FFA—free fatty acid, FPP—farnesyl diphosphate, GANG—disialoganglioside GD2, GGPP—geranylgeranyl diphosphate, GPP—geranyl pyrophosphate, HDL—High-Density Lipoprotein, IC_50_—concentration for 50% inhibition, IL—interleukin, IκB-α—inhibitory-κB protein, iNOS—inducible nitric oxide synthase, IPP—isopentenyl diphosphate, JNK—c-JUN N-terminal kinase, LDL—low-density lipoprotein, LOX—lipoxygenases, LPS—lipopolysaccharide, LXR—liver X receptor, Lyso-PC—lyso-phosphatidylcholine, Lyso-PL—lysophospholipid, MAPK—mitogen-activated protein kinase, MCI—mild cognitive impairment, MEP—2-C-methyl-D-erythritol 4-phosphate, MUFA—monounsaturated fatty acids, MVA—mevalonate, NF-κB—Nuclear factor-κB, NO—nitric oxide, NPD1—neuroprotectin D1, Nrf2—nuclear factor (erythroid-derived 2)-like 2, PA—phosphatidic acid, PC—phosphatidylcholine, PE—phosphatidylethanolamine, PG—phosphatidylglycerol, PGE2—prostaglandin-E2, PI—phosphatidylinositol, PKCδ—protein kinase C delta, PL—glycerophospholipids, PLG—plasmalogen, PS—phosphatidylserine, PS1—presenilin 1, PUFA—polyunsaturated fatty acids, ROS—reactive oxygen species, SC-CO_2_—supercritical carbon dioxide, SFA—saturated fatty acids, SM—sphingomyelin, SPL—sphingophospholipid, SREBP—sterol regulatory element binding protein, TAG—triacylglycerols, TNF-α—tumour necrosis factor-alpha, VDR—vitamin D receptor and VLDL—very low-density lipoprotein.
